# Transcriptional Programs and Regulators Underlying Age-Dependent and Dark-Induced Senescence in *Medicago truncatula*

**DOI:** 10.3390/cells11091570

**Published:** 2022-05-06

**Authors:** Kashif Mahmood, Ivone Torres-Jerez, Nick Krom, Wei Liu, Michael K. Udvardi

**Affiliations:** 1 Institute for Agricultural Biosciences, Oklahoma State University, Ardmore, OK 73401, USA; kashif.mahmood@okstate.edu (K.M.); ivone.torres.jerez@okstate.edu (I.T.-J.); ndkrom@noble.org (N.K.); wei.liu@unt.edu (W.L.); 2 Noble Research Institute, L.L.C., Ardmore, OK 73401, USA; 3 Department of Biological Sciences, BioDiscovery Institute, University of North Texas, Denton, TX 76201, USA; 4 Centre for Crop Science, The University of Queensland, St. Lucia, Brisbane, QLD 4072, Australia

**Keywords:** forage legumes, transcriptome analysis, transcription factors, heterologous expression

## Abstract

In forage crops, age-dependent and stress-induced senescence reduces forage yield and quality. Therefore, delaying leaf senescence may be a way to improve forage yield and quality as well as plant resilience to stresses. Here, we used RNA-sequencing to determine the molecular bases of age-dependent and dark-induced leaf senescence in *Medicago truncatula*. We identified 6845 differentially expressed genes (DEGs) in M3 leaves associated with age-dependent leaf senescence. An even larger number (14219) of DEGs were associated with dark-induced senescence. Upregulated genes identified during age-dependent and dark-induced senescence were over-represented in oxidation–reduction processes and amino acid, carboxylic acid and chlorophyll catabolic processes. Dark-specific upregulated genes also over-represented autophagy, senescence and cell death. Mitochondrial functions were strongly inhibited by dark-treatment while these remained active during age-dependent senescence. Additionally, 391 DE transcription factors (TFs) belonging to various TF families were identified, including a core set of 74 TFs during age-dependent senescence while 759 DE TFs including a core set of 338 TFs were identified during dark-induced senescence. The heterologous expression of several senescence-induced TFs belonging to NAC, WKRY, bZIP, MYB and HD-zip TF families promoted senescence in tobacco leaves. This study revealed the dynamics of transcriptomic responses to age- and dark-induced senescence in *M. truncatula* and identified senescence-associated TFs that are attractive targets for future work to control senescence in forage legumes.

## 1. Introduction

Senescence is the final stage of leaf development and enables plants to mobilize and re-use vital nutrients to support the growth of other vegetative or reproductive organs [1]. Age-dependent senescence is controlled by multiple internal and external factors including hormones, flowering-related signals, light quality and drought stress amongst others [2,3,4]. Although the timely onset and progression of senescence is important to ensure the reproductive success of plants through efficient resource allocation to seeds and vegetative storage organs, it comes at a cost to vegetative growth and/or quality. This trade-off is important in the context of forage crops in which vegetative tissues are the primary source of nutrients for animal production. Under less-than-ideal conditions, biotic and abiotic stresses activate senescence prematurely, reducing the quantity and quality of forage. On the other hand, delayed leaf senescence can lead to increased plant growth and quality, and resilience to environmental stresses such as drought [5,6,7,8].

A variety of mutants in different plant species are affected in senescence, including so-called “stay-green” mutants that exhibit delayed senescence [9,10]. Stay-green mutants are of two basic types, namely functional and non-functional in terms of photosynthesis and continued growth. Non-functional stay-green mutants are defective in the breakdown of chlorophyll and remain green even though chloroplasts are no longer photosynthetically active [9]. Functional stay-green mutants are more interesting from an agricultural point of view as they continue to grow under conditions that lead to senescence in the wild type [9]. This may be particularly desirable in forage species where the yield and quality of vegetative organs are important.

At the molecular level, the onset and progression of leaf senescence is associated with widespread changes in gene expression. Genes that are upregulated during leaf senescence, known as senescence-associated genes (SAGs), encode proteins that control senescence by regulating the expression of other genes, degrade macromolecules, or transport break-down products to other organs [4,11,12]. Transcription factors (TFs) play pivotal roles in the regulation of all biological processes including leaf senescence. A variety of TFs from different families, namely NAC (no apical meristem (NAM), ATAF, cup-shaped cotyledon (CUC)), WRKY (which contains the WRKY domain), myeloblastosis (MYB), basic helix-loop-helix (bHLH), C2H2 zinc finger, basic leucine zipper domain (bZIP) and APETALA2/ethylene responsive factor (AP2/ERF) have been implicated in the onset and progression of leaf senescence in different plant species [11,12,13,14,15,16,17,18,19,20,21,22,23,24,25,26]. Given that senescence can result from internal developmental as well as external environmental cues, it is of interest to compare the features of different senescence pathways to identify common and unique aspects. Such comparisons may enable us to target specific genes for plant breeding approaches that alter senescence to improve the yield and/or quality of forage and food crops.

The current study was undertaken to investigate and compare the transcriptional basis of age-dependent and stress-induced senescence in the model and pasture legume, *Medicago truncatula* Gaertn. (Fabaceae). A key objective of this work was to identify the transcriptional regulators of senescence as a prelude to future applied work to develop varieties with altered senescence programs that produce more high-quality forage under challenging environmental conditions.

## 2. Materials and Methods

### 2.1. Plant Growth Conditions and Tissue Sampling

*M. truncatula* (ecotype A17) seeds were scarified using concentrated sulfuric acid (H_2_SO_4_) for 8 min, followed by five washes of seeds with ddH2O. Scarified seeds were placed on 1% agar plates and stratified in the dark at 4 °C for three days. Plates were then transferred to a growth chamber (Conviron, Winnipeg, MB, Canada) overnight (16 h/8 h with 22 °C /18 °C for day/night) to promote seed germination. Germinated seedlings were transferred to soil pots (Metro-Mix^®^ 865) and then incubated in growth chambers under long day conditions (16 h/8 h with 22 °C/18 °C for day/night) at 200 µmol m^−1^ s^−2^ light intensity. For age-dependent senescence, all the main metameric leaves on *Medicago* plants were harvested at 10, 15, 21, 28, 35 and 42 days after sowing (DAS) and flash-frozen in liquid nitrogen. Four biological replicates were prepared for the age-dependent senescence assay, each being a pool of individual metameric leaves from six different plants. In *M. truncatula,* a metamer is defined as a growth/developmental unit comprising a main leaf (monofoilate or trifoliate) and associated axillary shoots that develops on the main stem [27]. For dark-induced senescence, plants were grown for 28 days under long day conditions (16 h/8 h with 22 °C /18 °C for day/night) at 200 µmol m^−1^ s^−2^ light intensity, and then exposed to continuous dark treatment for four consecutive days. Metameric (M3) leaves were harvested at 0 d, 1 d, 2 d, 3 d and 4 d of continuous dark and flash-frozen in liquid nitrogen. Frozen plant tissues were stored at −80 °C until processed. Three biological replicates were prepared for dark-induced senescence assay, each being a pool of individual metameric leaves from six different plants.

### 2.2. Determination of Chlorophyll Content

The chlorophyll content in the leaf samples were determined using a spectrophotometric method [28]. Leaves were ground in liquid nitrogen and 50 mg of leaf sample was incubated overnight in 80% acetone at 4 °C, followed by extraction of chlorophyll pigment three times. Two hundred microliters of supernatant were transferred to 96-well plates and absorbance was measured at 645 and 663 nm.

### 2.3. RNA Extraction, cDNA Synthesis and Quantitative RT-PCR Analysis

Total RNA from leaf samples was isolated using TRIZOL reagent (Life Technologies, Carlsbad, CA, USA), as described previously [29], then digested with RNase-free DNase I (Ambion, Austin, TX, USA) to remove DNA, and finally purified using RNeasy MinElute CleanUp kits (Qiagen, Hilden, Germany). RNA was quantified using a Nanodrop Spectrophotometer ND-100 (NanoDrop Technologies, Wilmington, DE, USA) and assessed for purity with a Bioanalyzer 2100 (Agilent Technologies, Wilmington, DE, USA). First-strand complementary DNA was synthesized by priming with oligo-dT20 (Qiagen), using Super Script Reverse Transcriptase III (Invitrogen GmbH, Karlsruhe, Germany) and following the manufacturer’s recommendations.

PCR reactions were carried out in an ABI PRISM 7900 HT Sequence Detection System (Applied Biosystems, Waltham, MA, USA). Five microliter reactions were set up in an optical 384-well plate containing 2.5 μL SYBR Green Power Master Mix reagent (Applied Biosystems), 15 ng cDNA and 200 nM of each gene-specific primer to amplify the target gene/s. The amplification of the target genes was achieved using the standard PCR protocol: 50 °C for 2 min; 95 °C for 10 min; 40 cycles of 95 °C for 15 s and 60 °C for 1 min, and SYBR Green fluorescence was measured continuously [30]. Melting curves were generated after 40 cycles by heating the samples to 95 °C for 15 s followed by cooling to 60 °C for 15 s. The transcript levels of target genes were normalized using the geometric mean of three housekeeping genes, *MtUbq* (Medtr3g091400), *MtPTB* (Medtr3g090960), and *MtUBC9* (Medtr7g116940).

### 2.4. Library Construction and RNA-Sequencing

One microgram of total RNA was used to generate the RNA-seq libraries using the TruSeq Stranded mRNA Library Prep Kit (Illumina Inc., San Diego, CA, USA) according to the manufacturer’s protocol. Prior to library construction, the RNA integrity and quality were assessed with TapeStation 4200 (Agilent Technologies), and only samples with an RNA integrity number (RIN) above nine were used. The size distribution of RNA-seq libraries was analyzed using TapeStation and the libraries were quantified using the Qubit 2.0 Fluorometer (ThermoFisher Scientific, Waltham, MA, USA) before being shipped to Novogene Inc., Beijing, China, for sequencing at 150 bp paired-end with an Illumina Hiseq2000 (Illumina Inc.). RNA-seq libraries were constructed from RNA samples representing three biological replicates for each timepoint.

### 2.5. Bioinformatics Analyses

To analyze the RNA-seq data, the total reads were processed before mapping to the *Medicago* gene sequences by employing Trimmomatic version 0.36 (http://www.usadellab.org/cms/?page=trimmomatic; accessed on 18 April 2021) to remove any low-quality bases and primer/adapter sequences. Reads less than 30 bases long after trimming were discarded, along with their mate pair. The trimmed reads were then mapped to version 4 of the *M. truncatula* genome, using HISAT2 version 2.1.0 with 24 threads and the default mapping parameters. Transcripts were assembled and quantified using Stringtie 2.1.2 with the default assembly parameters. The transcripts identified in all samples in the study were then compiled into a unified set of transcripts and compared with the reference genome’s set of transcripts using Stringtie’s “—merge” mode. Differential expression testing was performed using DESeq2.

Expression profiles in both datasets were identified using THE Short Time-Series Expression Miner (STEM, v1.3.13) program with default settings (*p* ≤ 0.05). The number of significant expression profiles was fixed at 40 (http://www.cs.cmu.edu/~jernst/stem; 21 October 2021) [31]. For Gene Ontology (GO)-term enrichment analysis, gene IDs of differentially expressed genes were fed into PlantRegMap to identify significant biological processes with *p* value ≤ 0.01 [32]. Redundant GO terms were removed using REVIGO with default parameters (http://revigo.irb.hr/ (accessed on 31 March 2022)) [33]. DiVenn analysis was performed online at https://divenn.tch.harvard.edu/ (accessed on 31 March 2022) to visualize and compare the DEGs obtained from the RNA-seq data representing different time points during age-dependent and dark-induced senescence as per Sun et al. [34].

### 2.6. Cloning of Senescence-Induced Transcription Factors and Transient Expression in Nicotiana Benthamiana

The coding regions of selected senescence-associated transcription factors (SA-TFs) were amplified from cDNA derived from senescing leaves of *M. truncatula* using gene-specific primers to amplify target SA-TFs (Supplementary Table S1) and were cloned into expression vector (MU71) under the control of the 35S CaMV promoter using the Gibson assembly method [35]. An expression vector was also constructed to overexpress GFP to be used as the negative control for senescence and positive control for gene expression. Expression vectors carrying SA-TFs (35S:SA-TFs) and GFP were transformed into *Agrobacterium tumefaciens*, GV2260 strain via electroporation. GV2260 cultures carrying plasmids for selected SA-TFs (final OD_600_, 0.5) and GFP were mixed with GV2260 culture harboring 35S:TBSV p19 plasmid (final OD_600_, 0.1) in the infiltration buffer [10 mM MeS (pH = 5.8) and 200 μM acetosyringone], and incubated at 28 °C with gentle shaking for ~3 h. P19 protein is encoded by Tobacco Bushy Shunt Virus genome and is a known suppressor of posttranscriptional gene silencing (PTGS). Abaxial sides of the 4th and 5th leaves of *N. benthamiana* plants (4-week-old) from the top were gently infiltrated with *Agrobacterium* cultures using a 1 mL syringe and were grown under long day conditions (16 h/8 h with 22 °C/18 °C for day/night) at 200 µmol m^−1^ s^−2^ light intensity. The infiltrated tobacco leaves were monitored daily for the emergence of senescence symptoms, and were finally harvested seven days after infiltration, photographed, and scanned under UV light for GFP expression.

### 2.7. Statistical Analysis

Quantitative RT-PCR data were analyzed using the two-tailed Student’s t-test in MS Excel. Chlorophyll data (total Chl, Chla and Chlb) across different time points of metameric leaves were analyzed by one-way ANOVA LSD test (*p* ≤ 0.5) using the GraphPad Prism software (La Jolla, CA, USA).

## 3. Results

### 3.1. Characterization of Age-Dependent Leaf Senescence in M. truncatula

**Figure 1:**
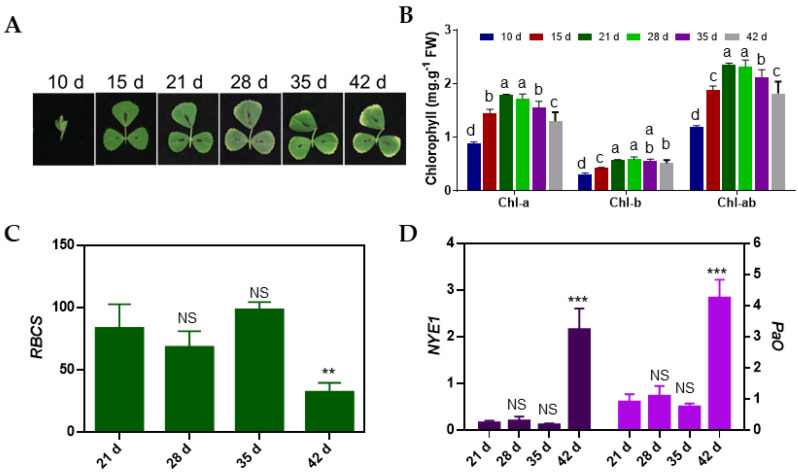
Physiological and molecular analysis of leaf growth and senescence. (**A**) Phenotypic analysis of leaf growth and senescence in M3 leaves at 10 d, 15 d, 21 d, 28 d, 35 d and 42 d after sowing. (**B**) Chlorophyll content (chla, chlb and total chlorophyll (chl-ab) in M3 leaf at 10 d, 15 d, 21 d, 28 d, 35 d and 42 d. Data represent mean values (±SD; *n* = 4), and were analyzed using one-way ANOVA LSD test (*p < 0.05*). Bars with different letters are statistically different from each other. (**C**) Expression analysis of photosynthesis-associated gene (PhAG, *RBCS*) at 21 d, 28 d, 35 d and 42 d. (**D**) Expression analysis of senescence-associated genes (SAGs, *NYE-1* and *PaO*) at 21 d, 28 d, 35 d and 42 d. Data in (**C**) and (**D**) represent mean values (±SD; *n* = 4) and were analyzed using Student’s *t*-test (NS; not significant, ** *p* < 0.01, *** *p* < 0.001) against 21 d time point.

To characterize the onset and progression of leaf senescence in *M. truncatula*, we closely monitored the leaf growth and senescence patterns and determined the chlorophyll concentration in all main metameric leaves of plants. Different leaves accumulated different levels of chlorophyll by a given time due to differences in their age. For example, the chlorophyll concentration of M1 monofoliate leaves reached its peak by 15 DAS, but in M2 and M3 leaves, the first and second main trifoliate leaves, respectively, the chlorophyll concentration was highest at 21 DAS (Supplementary Figure S1). These sampling time-points allowed us to observe the leaf growth and senescence in all main metameric leaves until 42 DAS. We selected M3 trifoliate leaves to investigate the details of leaf development and senescence because the M3 leaves had clearly begun senescence by 42 DAS and the plants had not begun to flower by that time. The chlorophyll concentration in M3 leaves at 28 DAS was comparable to 21 DAS, suggesting that M3 leaves were mature and fully functional at 21 DAS (Figure 1A,B). M3 leaves began to exhibit senescence symptoms at 35 DAS at the leaf margin, which expanded by 42 DAS (Figure 1A). Senescence symptoms in M3 leaves coincided with a significant decrease in chlorophyll concentration at 35 and 42 DAS compared to earlier time points (Figure 1B). We analyzed the expression of homologs of well-known *Arabidopsis* photosynthesis-associated gene (PhAG), RUBISCO small subunit (RBCS), and senescence-associated genes (SAGs), encoding the chlorophyll degradation proteins such as non-yellowing-1 (NYE-1) and Pheophorbide a Oxygenase (PaO) in *M. truncatula* from 21 DAS onwards. The qRT-PCR analysis revealed that the expression of *RBCS* decreased while the expression of *NYE-1* and *PaO* increased at 42 DAS in M3 leaves (Figure 1C,D), indicating that the senescence process was active in older M3 leaves.

### 3.2. Transcriptomic Analysis of Age-Dependent Senescence

**Figure 2:**
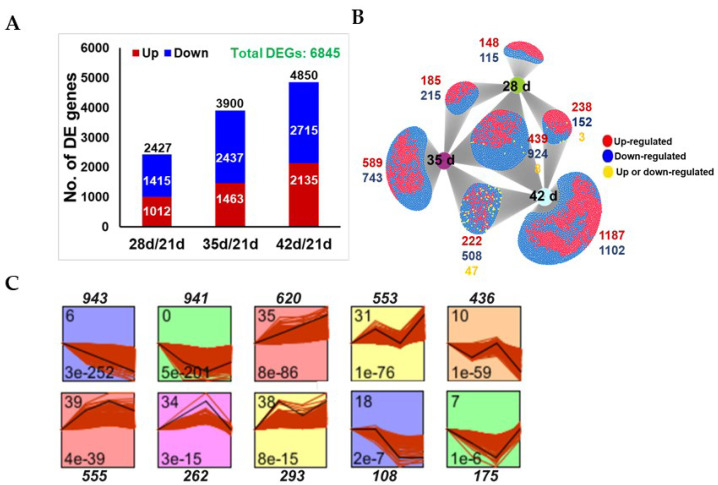
Transcriptomic analysis of age-dependent senescence. (**A**) Numbers of differentially expressed genes (DEGs) (*Padj* ≤ 0.05, Log2FC ± 1) in M3 leaves at day 28, 35 and 42 vs. day 21, calculated from the FPKM values from three biological replicates for each time point. (**B**) Venn diagram showing overlap among DEGs in M3 leaves at 28, 35 and 42 DAS vs. 21 DAS, using DiVenn (https://divenn.tch.harvard.edu; accessed on 30 March 2022). (**C**) STEM clustering of DEGs exhibiting similar expression patterns at 21, 28, 35 and 42 DAS. The numbers outside the boxes in (**C**) represent gene numbers. The numbers in the upper left corner inside the boxes in (**C**) indicate the profile number, and in the lower left corner, the *p* values.

As M3 leaves had a maximum chlorophyll concentration at 21 DAS, which gradually declined at later time points (35 d and 42 d), we selected four time points for transcriptome profiling; 21 d, 28 d, 35 d and 42 d. Transcriptomic data were mapped to the Medicago MT.4 genome assembly and compared to 21 d levels to identify the differentially expressed (DE) genes, with transcript levels significantly higher or lower that those at 21 d. Among the 40,687 expressed genes in the dataset, a total of 6845 (16.82%) genes were found to be differentially expressed genes (DEGs) at different time points after 21 d (Padj ≤ 0.05 and Log2FC ≥ ±1) (Supplementary Dataset S1, S2). These included 2427, 3900, and 4850 DEGs at 28 d, 35 d and 42 d, respectively, showing that the number of DEGs increased with leaf age. Overall, more genes were found to be downregulated at each time point than upregulated compared to the baseline at 21 d (Figure 2A). DiVenn analysis [34] revealed the number and identification of DEGs shared between two or more growth stages, as well as stage-specific DEGs. A core set of DEGs (1371 DEGs) among all growth phases was identified that could potentially be involved in the onset and progression of leaf senescence in *M. truncatula* (Figure 2B and Supplementary Dataset S2). Interestingly, putative orthologs of several of these genes are induced in aging leaves of *A. thaliana*. For example, the induced set of core genes included Medtr4g081870 (NAC TF) and Medtr5g043880 (WRKY TF) whose closest homologs in *Arabidopsis*, AtNAP and WRKY57, respectively, are known to promote leaf senescence [36,37]. Similarly, other orthologs of *Arabidopsis* senescence modulating genes such as Medtr8g059170 (ANAC072/RD26), and Medtr2g068880 (JUNGBRUNNEN1, JUB1) were also induced in *M. truncatula* at 42 DAS compared to 21 DAS, (Supplementary Dataset S2). The induction of these genes coincided with visible senescence symptoms observed in the M3 leaves at 42 DAS but not 21 DAS.

We performed the cluster analysis of all DEGs using Short Time-Series Expression Miner (STEM, [31]) to identify the predominant expression profiles associated with M3 leaf development and senescence. In total, we identified five significant and distinct expression profiles for the upregulated genes and five for the downregulated genes, using the default parameters of STEM (Figure 2C and Supplementary Figure S2 and Dataset S3). For example, profile # 6 was enriched in genes that exhibited a consistent decreasing trend during senescence including Medtr6g043600 (chlorophyll A/B binding protein, involved in chlorophyll biosynthesis), Medtr2g090675 (Golden2-type transcription factor-GLK1, involved in chloroplast development) and Medtr8g086600 (cellulose synthase A catalytic subunit 8, involved in cellulose biosynthesis). Genes in profile # 0 exhibited decreasing levels of transcripts at 28 DAS and 35 DAS that stabilized or slightly increased at 42 DAS compared to 21 DAS (Figure 2C and Supplementary Dataset S3) such as Medtr1g064090 (phenylalanine ammonia-lyase 1, involved in phenylpropanoid pathway) and Medtr4g129630 (ECERIFERUM 3, involved in fatty acid biosynthesis). Similarly, genes in profile # 35 exhibited an increasing trend in transcript levels throughout senescence such as Medtr2g060350 (a pectin lyase, involved in cell degradation) and Medtr7g114240 (superoxide dismutase CU/ZN, involved in removal superoxide radicals). Profile # 39 was enriched with genes that were upregulated at 28 DAS and 35 DAS but not at 42 DAS compared to 21 DAS, such as Medtr6g005630 (a pectin lyase involved in pectin/cell wall degradation) and Medtr4g086190 (an AP2/ERF TF) (Figure 2C and Supplementary Figure S2 and Dataset S3).

### 3.3. Gene Ontology Enrichment Analysis of Medicago DEGs

To identify the biological processes involved in leaf senescence, we performed the Gene Ontology (GO) enrichment analysis of DEGs. Enriched GO-terms and the associated genes for each expression profile were determined using PlantRegMap (*p* ≤ 0.01) [32], followed by the removal of redundant GO-terms using REVIGO [33]. Lists of total and reduced GO-terms and associated SAGs for each expression profile are presented in the Dataset S4 and the top four GO-terms per expression profile are shown in Supplementary Figure S3. Genes in profile #0 whose expression significantly decreased at 28 d and 35 d were largely associated with defense responses, cell communication and stress response, suggesting that defense and stress responses weaken with age. Genes in profile #6 whose expression decreased continuously at 28 d, 35 d and 42 d were associated with cell wall biogenesis, carbohydrate metabolism and phenylpropanoid metabolic pathways. Similarly, genes in profile #7 were involved in photosynthesis and auxin response—processes that are typically associated with active leaf growth (Supplementary Figure S3 and Dataset S4). Moreover, genes downregulated between 28 d and 35 d in profile 18 were related to cell wall modifications such as pectin metabolism (Supplementary Figure S3 and Dataset S4). Thus, key processes underlying leaf growth and vigor were downregulated alongside senescence. In contrast, the expression profiles representing induced SAGs were enriched in biological processes associated with catabolism and transport. For example, profile #31 with genes exhibiting the greatest induction at 42 d was enriched in genes involved in porphyrin catabolism, amino acid and peptide transport, as well as response to external stimuli (Supplementary Figure S3 and Dataset S4). Profile #34 contained genes that were gradually induced until 35 d but then strongly repressed. These genes are involved in protein synthesis and nitrogen metabolism, suggesting that these biological processes are maintained early during senescence but shut down at later stages (i.e., by 42 DAS). Profile # 35, characterized by a consistent increase in gene expression, was enriched in terpenoid (mono-) biosynthesis, branched chain amino acid catabolism, and fatty acid beta-oxidation. Protein degradation is one of the major pathways activated during leaf senescence to facilitate the turnover of proteins and the mobilization of amino acids. In this context, the upregulated profiles contained multiple members of protein degrading enzymes such as cystein proteases, serine proteases and 26S proteasome pathway, including E2 (ubiquitin-conjugating enzyme) and E3 ligases (RING type and FBOX ligases) (Supplementary Dataset S3, S4). These included Medtr4g072340 (a cysteine protease), Medtr4g015600 (RING type) and Medtr1g029500 (FBox protein) whose orthologs in *Arabidopsis* were also induced during leaf senescence [11].

### 3.4. Transcription Factors during Age-Dependent Senescence

**Figure 3:**
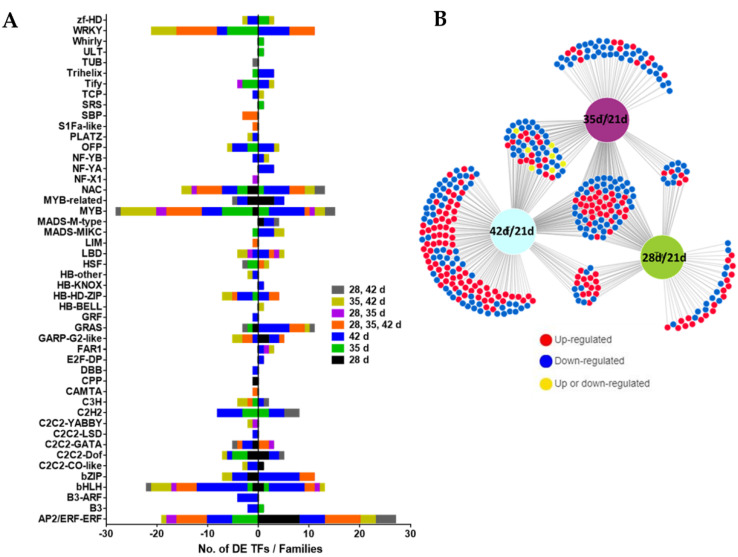
Transcriptional regulation of age-dependent senescence. (**A**) Graphical representation of DE TF families at 28, 35, 42 DAS vs. 21 DAS. (**B**) DiVenn analysis showing overlap among DE TFs in M3 leaves at 28, 35, 42 DAS vs. 21 DAS.

We identified 391 DE TFs in our leaf development RNA-seq time-series data, including 142 TFs (68 up and 74 downregulated) at 28 DAS, 195 TFs (65 up, 130 down) at 35 DAS, and 294 TFs (143 up, 151 down) at 42 DAS compared to 21 DAS. Venn diagram analysis identified a subset of 74 TFs (31 upregulated and 43 downregulated) that were differentially expressed at all time points during leaf development, as well as others that were differentially expressed at specific stages (Figure 3A,B and Supplementary Dataset S2). These TFs belong to 48 different families of transcription factors. TF families such as AP2/ERF, bHLH, bZIP, MYB/MYB-related, GRAS, NAC and WRKY, were most overrepresented and many genes from these TF families were differentially expressed at one, two or all time points during leaf development compared to 21 d (Figure 3A,B and Supplementary Dataset S2). Additionally, we analyzed all the upregulated and downregulated expression profiles identified through STEM analysis to see the frequency and types of TFs present in each expression profile and to determine whether the identified TFs could be related to biological processes enriched in that particular expression profile. Interestingly, the upregulated expression profiles were enriched with members from AP2/ERF, bZIP, GRAS, MADS-MIKC, MADS-M-type, NAC and WKRY TF families (Table 1), and these profiles are enriched in biological processes related to protein ubiquitination, oxidation–reduction processes, amino acid metabolism, and responses to external stimuli (Supplementary Figure S3 and Dataset S3, S4).

### 3.5. Characterization of Dark-Induced Leaf Senescence in M. truncatula

**Figure 4:**
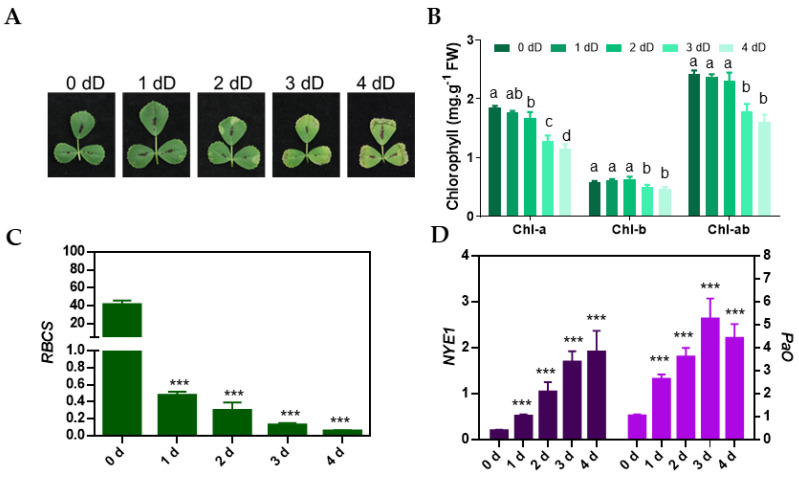
Physiological and molecular analysis of dark-induced leaf senescence. (**A**) Phenotype of M3 trifoliate leaves from 28-day-old plants at 0 d, or after an additional 1 d, 2 d, 3 d or 4 d of dark treatment (dD—days of dark treatment). (**B**) Chlorophyll concentration (Chl-a, Chl-b and total chlorophyll, Chl-ab) in M3 trifoliate leaf at 0 dD, 1 dD, 2 dD, 3 dD and 4 dD. Data represent mean values (±SD; *n* = 3) and were analyzed using one-way ANOVA LSD test (*p <* 0.05) and bars with different letters in (**B**) are statistically not similar to each other. Expression analysis of (**C**) photosynthesis-associated genes (PhAG, *RBCS*) and (**D**) senescence-associated genes (SAGs, *NYE-1* and *PaO*), at 0 dD, 1 dD, 2 dD and 3 dD. Data in (**C**) and (**D**) represent mean values (±SD; *n* = 3), and were analyzed using Student’s *t*-test (*** *p* < 0.001) against 0 dD time point.

To characterize the physiological and molecular changes associated with dark-induced senescence, *M. truncatula* plants at 28 DAS were exposed to continuous dark for four days, and M3 leaves were harvested at 0 d, 1 d, 2 d, 3 d, and 4 d of dark treatment to analyze the chlorophyll content. At 28 DAS, M3 leaves were green and showed no symptoms of senescence (leaf yellowing). Measurements of chlorophyll pigment revealed no significant difference in the total chlorophyll concentration until two days after dark treatment (Figure 4A,B), although the Chl-a concentration decreased slightly. However, after this time point, a sharp decline in chlorophyll concentration occurred, accompanied by chlorosis and leaf wilting on the third and fourth days of dark treatment (Figure 4A,B). Measurements of the transcript levels of a PhAG and SAGs in leaf samples by qRT-PCR revealed a strong downregulation of *RBCS* and significant induction in the expression of *NYE-1* and *PaO* (chlorophyll degradation genes), respectively, in response to extended dark treatment (Figure 4C,D). This indicated that the dark treatment invoked a strong molecular response in *M. truncatula* before its impact was visible at the physiological level. We used the same RNA samples in RNA-seq analysis to determine the impact of continuous dark treatment on the expression of all genes.

### 3.6. Transcriptomic Profiling of Dark-Induced Senescence in M. truncatula

**Figure 5:**
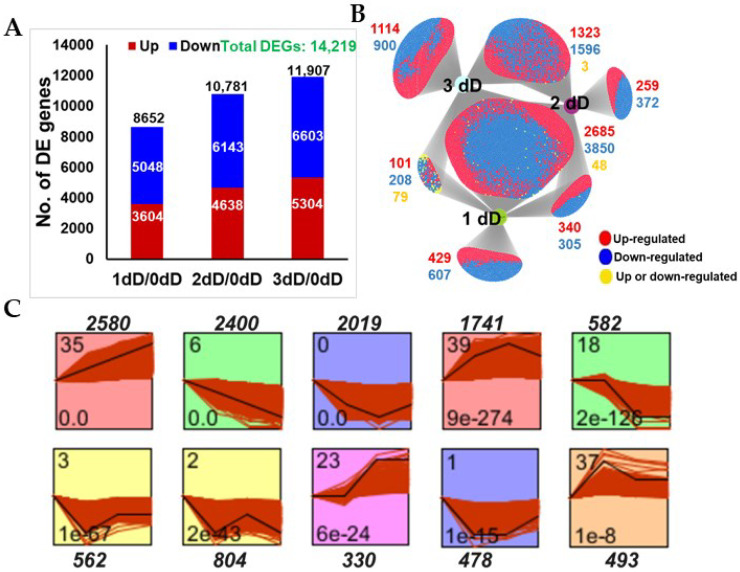
Transcriptomic analysis of dark-induced senescence in *M. truncatula*. (**A**) Number of differentially expressed genes (DEGs) (*Padj ≤* 0.05, Log2FC ± 1) at 1 dD, 2 dD, 3 dD vs. 0 dD (dD—day after dark treatment), calculated from the FPKM values from three biological replicates for each time point. (**B**) Venn diagram showing overlap among DEGs at 1 dD, 2 dD, 3 dD vs. 0 dD using DiVenn. (**C**) STEM clustering of DEGs exhibiting similar temporal expression patterns at 0 dD, 1 dD, 2 dD and 3 dD. The numbers outside the boxes in (**C**) represent the gene numbers. The numbers in the upper left corner inside the boxes in (**C**) indicate the profile number, and in the lower left corner, the *p* values.

Dark treatment of M3 leaves had a greater impact than the developmental time on the global gene expression associated with senescence. In total, we found 14,219 genes (~34.95%) to be differentially expressed at least at one time point (Log2FC ±1, Padj ≤ 0.05) during dark treatment, compared to 0 day (Figure 5A and Supplementary Dataset S5). After only one day of dark treatment, 8652 (3604 upregulated, 5048 downregulated) genes were differentially expressed, and the number of DEGs increased with treatment duration over the following days (Figure 5A and Supplementary Dataset S5). More genes were downregulated by dark treatment at each time point than were upregulated; 3604 genes were upregulated and 5048 genes were downregulated at 1 d, 4638 were upregulated and 6143 were downregulated at 2 d, and 5304 were upregulated and 6603 were downregulated at 3 d of dark treatment. The comparison of transcript profiles using the DiVenn tool [34] showed that 6583 genes (2685 upregulated, 3650 downregulated and only 48 variably regulated) were differentially expressed at all times points after the start of dark treatment. This comparison also identified sets of genes specific to only one time point (specific to day 1, 2 or 3) or shared between two time points, such as between 1 d and 2 d; 2 d and 3 d, and 1 d and 3 d (Figure 5B and Supplementary Dataset S5). Interestingly, we identified several putative orthologs of *Arabidopsis* SAGs that are known to promote leaf senescence. These included Medtr5g041940 and Medtr4g081870 (AtNAP/ANAC029); Medtr8g059170 and Medtr2g079990 (ANAC072/RD26); Medtr8g096730, Medtr7g028710 and Medtr7g028415 (AtWRKY75); Medtr1g418545 (ACBP3, Acyl-coA-binding protein 3, involved in lipid degradation) [36,38,39]. Moreover, Medtr4g080700 and Medtr4g107930, putative orthologs of AtSAG12, a marker gene for leaf senescence, were also induced in our dark-treated M3 leaves.

Then, we analyzed all DEGs using STEM with default settings to identify upregulated and downregulated gene profiles. In total, six significant expression profiles were identified for downregulated genes exhibiting different temporal patterns and four expression profiles for genes upregulated during dark treatment. For example, genes in profile # 6 exhibited a continued decrease in their expression during dark treatment, while in profile # 35, the genes exhibited a continued increase in their expression (Figure 5C and Supplementary Figure S4 and Dataset S6).

### 3.7. GO-Term Enrichment Analysis of Dark-Induced DEG Clusters

GO-term enrichment analysis revealed that biological processes associated with plant growth and development were strongly enriched in all downregulated gene clusters. For example, genes in profiles 0, 1, and 3 that showed a reduction in their expression at 1 d and 2 d were involved in biological processes related to photosynthesis, carbohydrate metabolism, and response to abiotic stresses such as temperature stress (Supplementary Figure S5 and Dataset S7). Genes in profile # 6 which exhibited a continued decrease in their expression from day 0 to day 3 during dark treatment were related to protein translation, ribosome biogenesis, protein folding and defense signaling response. Similarly, profile 18 was predominantly enriched with genes involved in phosphorus metabolism, protein phosphorylation/modification and plant defense response (Supplementary Figure S5 and Dataset S7). For upregulated gene clusters, profile 23 showed increased expression after 1 d of dark treatment and was enriched in genes involved in ion homeostasis, circadian rhythm, and the regulation of response to red and far-red light. Profile # 35, which had the greatest number of upregulated genes and exhibited an increasing trend in transcript levels at 1 d, 2 d and 3 d, was enriched in biological processes related to leaf aging and senescence, autophagy, response to biotic and abiotic stresses, including important plant stress hormones such as abscisic acid. Genes in profile # 37 were induced maximally by 1 d and were enriched in biological processes associated with pyrimidine biosynthesis and response to wounding. Genes in profile # 39 had an increased expression until 2 d and then decreased and were predominantly enriched in biological processes related to carboxylic acid and branched chain amino acids catabolism, responses to biotic and abiotic stimuli and ROS metabolic processes (Supplementary Figure S5 and Dataset S7).

### 3.8. Transcription Factors Responding to Dark Treatment

**Figure 6:**
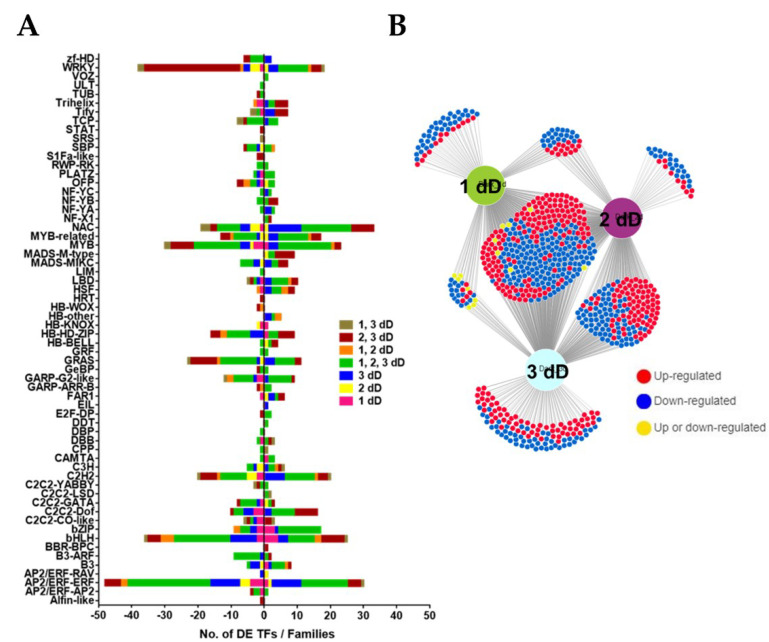
Transcriptional regulation of dark-induced senescence. (**A**) Graphical representation of differentially expressed transcription factor families at 1 d, 2 d, 3 d vs. 0 d of dark treatment (dD). (**B**) Venn diagram analysis showing overlap among differentially expressed transcription factors in M3 leaves at 1 d, 2 d, 3 d vs. 0 d of dark treatment using DiVenn.

We identified 449 dark-responsive TF genes (198 up- and 251 downregulated) at 1 d, 567 TFs (274 up, 293 down) at 2 d, and 645 TFs (324 up and 321 down) at 3 d, compared to the 0 d of dark treatment. In total, 759 TFs belonging to 60 TF families were differentially expressed at one or more time points during dark treatment (1 d, 2 d and 3 d vs. 0 d) (Figure 6A and Supplementary Dataset S5). Venn diagram analysis revealed some overlap among these DE TFs at different time points (Figure 6A). Moreover, a sub-set of 338 TFs (160 induced and 171 repressed and 7 variably regulated) were differentially expressed at all the time points during dark treatment, compared to day 0, and are potentially involved in both the initiation and progression of dark-induced leaf senescence in *M. truncatula* (Figure 6A and Supplementary Dataset S5). The majority of DE TFs belonged to AP2/ERF, bHLH, bZIP, C2H2, GRAS, MYB/MYB-related, NAC and WRKY, C2C2-Dof, HSF and MADS-box (MIKC and M-type) TF families (Figure 6B and Supplementary Dataset S5).

We pursued up- or downregulated expression profiles to see whether certain TFs families in these profiles were associated with specific biological functions (GO-terms). Interestingly, we found that downregulated expression profiles were mainly enriched with TF families normally associated with growth and photosynthesis such AP2/ERF, bHLH, GATA, GARP-G2-like, and MYBs. On the other hand, upregulated expression profiles were enriched with TFs implicated in the regulation of stress responses, including leaf senescence. These included bZIP, bHLH, MYB, NAC, and WRKY TF families (Table 2).

### 3.9. Comparison of Age-Dependent and Dark-Induced Transcriptomic Profiles

**Figure 7:**
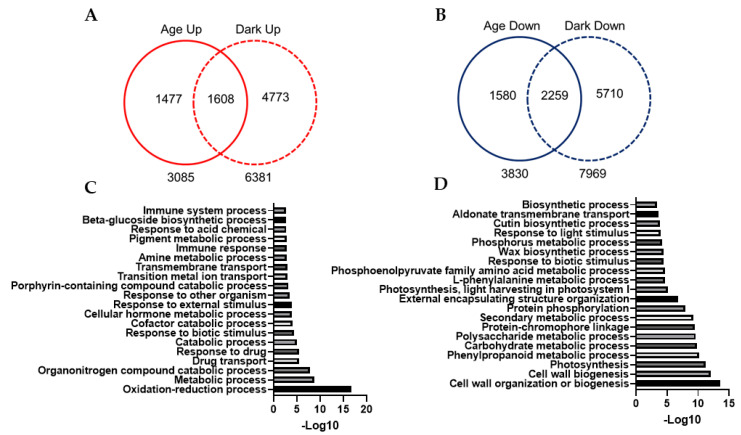
Comparison of age-dependent and dark-induced transcript profiles. (**A**) Venn diagram showing overlap among all the DE upregulated genes in age-dependent and dark-induced leaf senescence. (**B**) Venn diagram showing overlap among all the DE downregulated genes in age-dependent and dark-induced leaf senescence. (**C**) GO-term enrichment analysis of common upregulated genes during age-dependent and dark-induced leaf senescence. (**D**) GO-term enrichment analysis of common downregulated genes during age-dependent and dark-induced leaf senescence.

To better understand the similarities and differences between age-dependent and dark-induced senescence in *Medicago*, we compared the transcriptome profiles of M3 leaves undergoing normal developmental and dark-induced senescence. The comparison between all the upregulated genes identified during age-dependent (3085 genes) and dark-induced senescence (6381 genes) revealed an overlap of 20.5% (1608) for the upregulated genes between the two datasets. Similarly, the comparison between all the downregulated genes identified during age-dependent (3839 genes) and dark-induced senescence (7969 genes) revealed an overlap of 23.7% (2257) for the downregulated genes between the two datasets (Figure 7A,B and Supplementary Dataset S8). We then separately analyzed the shared upregulated and downregulated genes using GO-term enrichment analysis to determine the biological processes common to age-dependent and dark-induced senescence pathways. The downregulated genes common to both pathways were enriched in biological processes related to plant growth and development such as photosynthesis, cell wall biogenesis, phenylpropanoid metabolism, cutin and wax biosynthesis, whereas shared upregulated genes were found to be involved in the biological processes related to the degradation of macromolecular structures and remobilization such as oxidation–reduction processes and amino acid, carboxylic acid and chlorophyll catabolic processes, and responses to external and biotic stimuli (Figure 7C,D and Supplementary Dataset S8). We also performed GO-term enrichment analysis for upregulated and downregulated genes specific to age-dependent or dark-induced transcriptomes (Figure 7A,B and Supplementary Dataset S8) to identify the biological processes that may be unique to one or the other. The Venn diagram analysis of the GO terms for each gene category (upregulated or downregulated) specific to each transcriptomic profile revealed little overlap, suggesting that these specific sets of upregulated and downregulated genes are involved in different biological processes (Supplementary Figure S6A,D and Dataset S8). For example, the downregulated genes specific to dark-treatment encoded biological processes such as protein translation, amino acid biosynthesis, ribosome biogenesis, and mitochondrial mRNA processing, in contrast to age-specific senescence where genes regulating protein translation and other growth-related biological processes were still actively transcribed (Figure S6B,E,F and Dataset S8). Interestingly, genes downregulated specifically during age-dependent senescence were also enriched in biological processes related to plant growth such as photosynthetic electron transport, cell wall biosynthesis and microtubule-based movement (Supplementary Figure S6E and Dataset S8), suggesting the involvement of a unique or additional set of genes specific to age-dependent senescence to regulate the similar growth-related biological processes that were commonly downregulated during age-dependent and dark-induced senescence. Dark-specific upregulated genes were enriched in biological processes related to autophagy, aging/senescence, cell death and responses to biotic and abiotic stresses (Supplementary Figure S6C and Dataset S8), suggesting a drastic activation of the senescence programs that are normally associated with stress-induced senescence.

### 3.10. Comparison of Age-Dependent and Dark-Induced Leaf Senescence Transcriptomic Profiles between Medicago and Arabidopsis

**Figure 8:**
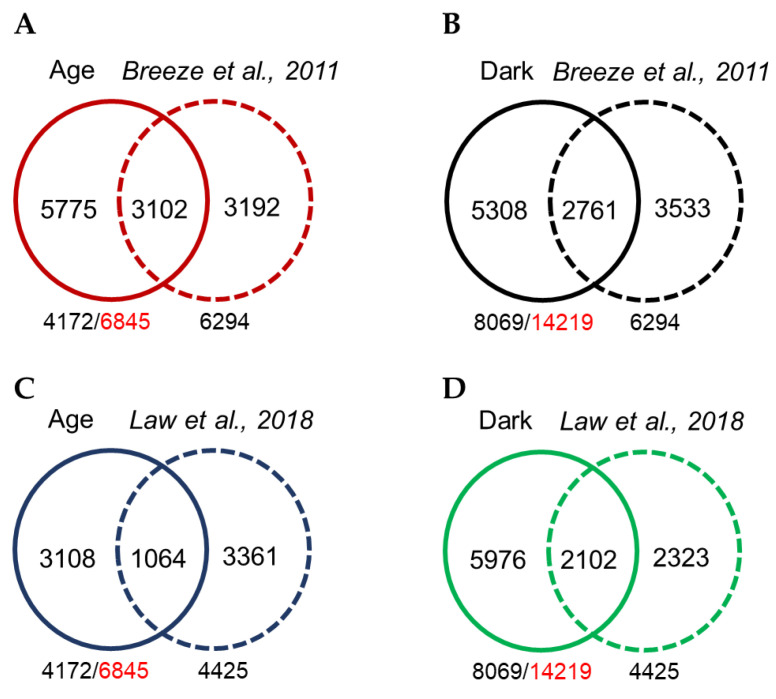
Comparison of *M. truncatula* and *A. thaliana* age-dependent and dark-induced transcriptomic profiles. (**A**) Comparison of *M. truncatula* age-dependent transcriptomic data with that of *A. thaliana* reported by Breeze et al., 2011. (**B**) Comparison of *M. truncatula* dark-induced transcriptomic data with that of *A. thaliana* reported by Breeze et al., 2011. (**C**) Comparison of *M. truncatula* age-dependent transcriptomic data with *A. thaliana* dark-induced reported by Law et al., 2018. (**D**) Comparison of *M. truncatula* dark-induced transcriptomic data with *A. thaliana* dark-induced transcriptomic data reported by Law et al., 2018.

Out of the 6845 DEGs associated with age-dependent senescence in *M. truncatula*, we identified 4172 homologous genes in the *A. thaliana* genome. To determine the extent of conservation in the leaf senescence processes in *M. truncatula* and *A. thaliana*, we compared our lists of *Arabidopsis* genes matching *Medicago* age-dependent and dark-induced DEGs with transcriptomic data from *Arabidopsis* for age-dependent leaf senescence [11]. The *Arabidopsis* study identified 6294 DEGs associated with different stages of leaf development including leaf senescence [11]. We found 15.7% (1563 genes) similarity in the age-dependent transcriptomic datasets of both plant species (Figure 8A). On the other hand, 23.8% (2671 genes) similarity was found between the *Medicago* dark-induced and *Arabidopsis* age-dependent transcriptomic datasets (Figure 8B). These results suggest that despite some level of conservation, there are substantial differences in regulation of senescence in these two species. We also compared our age-dependent and dark-induced transcriptomic profiles with the recently published transcriptomic profile of dark-treated *Arabidopsis* [26]. This study identified 4425 DEGs responding to dark treatment. Interestingly, we found 14.1% (1064 genes) similarity between *Medicago* age-dependent and *Arabidopsis* dark-treated DEGs, and 20.2 % (2102 genes) similarity between *Medicago* dark- and *Arabidopsis* dark-induced DEGs (Figure 8C,D). This further confirms that senescence in the two species shares many genes in common, as well as many genes that are unique to one species or the other.

### 3.11. Identification of Senescence Promoting Transcription Factors Using a Transient Expression System

**Figure 9:**
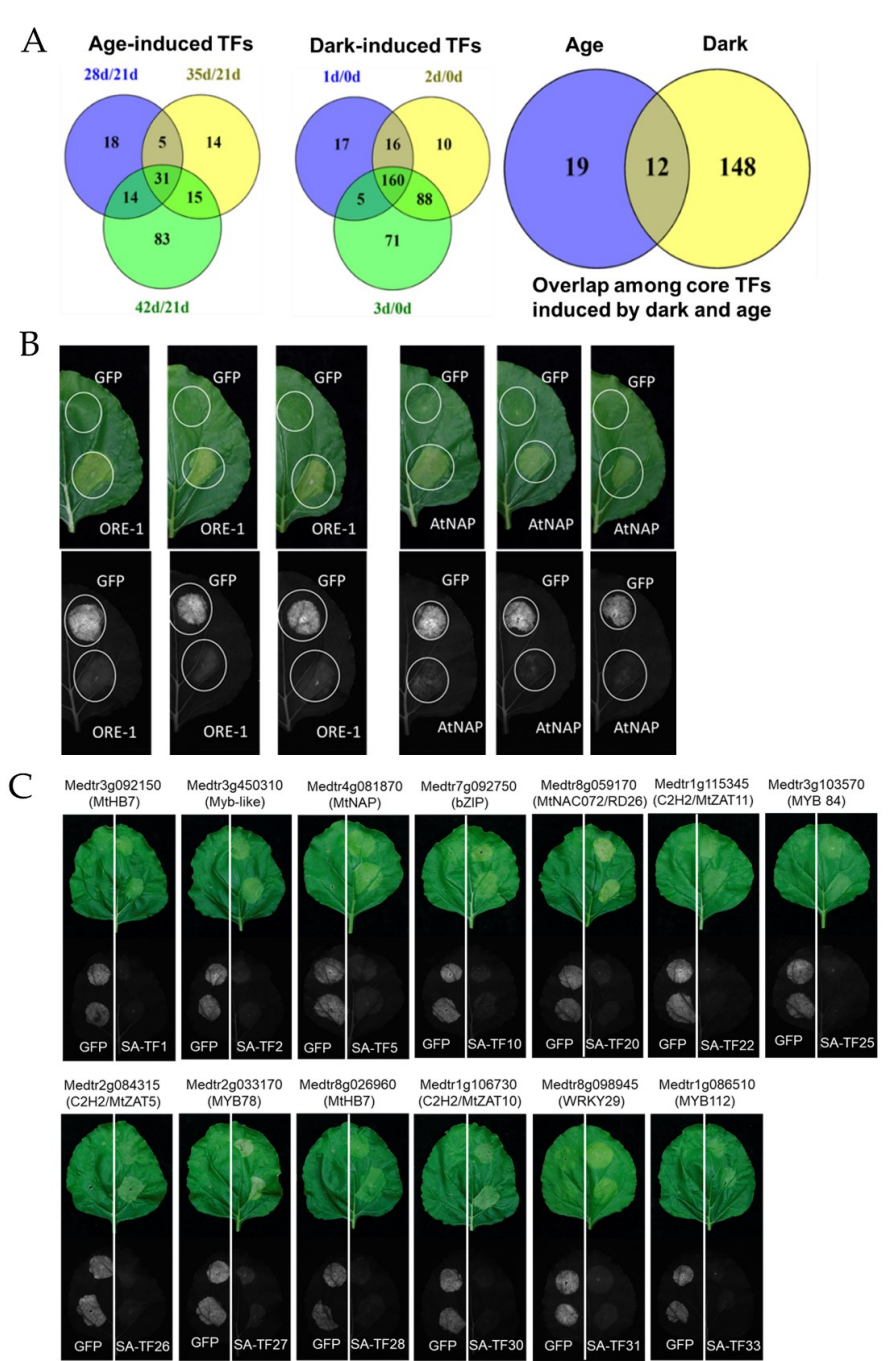
Transient expression of putative senescence-associated transcription factors (SA-TFs). (**A**) Venn diagrams of senescence-induced TFs at different time points in age-dependent and dark-induced senescence. (**B**) Transient overexpression of ORE-1 and AtNAP (35S:TFs) in tobacco leaves through the agroinfiltration method. Expression of GFP under 35S promoter was used as a positive control for gene expression and negative control for senescence induction. (**C**) SA-TFs were cloned into expression vectors under 35S CaMV promoter and transiently expressed in tobacco leaves using the agroinfiltration method. Leaves were photographed six to seven days after infiltration.

We carried out these transcriptomic studies to identify key TFs involved in the promotion of leaf senescence (SA-TFs), with the goal of harnessing their potential to improve biomass yield, nutritional quality and plant resilience to stress, via genome editing to delay leaf senescence. We identified many TFs that were induced during age-dependent and dark-induced leaf senescence (Figure 9A). Since the generation or isolation of homozygous mutants in candidate TFs and their characterization can take years, we employed *Agrobacterium*-mediated transient gene expression in tobacco leaves for rapid screening of *Medicago* SA-TFs. This method has been extensively used in plant biology research to study protein subcellular localization, DNA–protein and protein–protein interactions, screening genes involved in suberin biosynthesis and chlorophyll degradation, amongst other things [40,41,42,43,44].

To determine whether this system could be used for the rapid screening of novel SA-TFs, we first analyzed two well-known senescence-promoting NAC TFs, ORE-1 and AtNAP, from *A. thaliana*, by transiently expressing them under the control of the 35S CaMV constitutive promoter (35S:NACs) in tobacco leaves. We used 35S:GFP as a positive control for transgene expression and as a negative control for senescence induction. We found that GFP was visibly expressed in tobacco leaves but did not induce any senescence symptoms. However, the expression of either ORE-1 or AtNAP strongly induced senescence symptoms, i.e., leaf yellowing/loss of chlorophyll in the infiltrated zones of tobacco leaves (Figure 9B). We then overexpressed 26 different TFs that were induced in our age-dependent and dark-induced senescence transcriptomes in *M. truncatula*. These included 9 of the 12 common core TFs induced at all the time points during age-dependent and dark-induced senescence, 9 core TFs specific to age-dependent senescence, and 8 core dark-induced TFs (Supplementary Table S2). Interestingly, all these 26 TFs were upregulated in M3 leaves at the last time points (42 vs. 21 DAS and 3 dD, vs. 0 dD) harvested for age-dependent and dark-induced senescence (Figure 9A,B and Supplementary Dataset S2, S5). At least 13 out of 26 TFs were found to induce senescence/cell death-like symptoms when transiently overexpressed in tobacco leaves, including the orthologs of AtNAP and AtNAC072, Medtr4g081870 and Medtr8g059170, respectively (Figure 9C and Supplementary Figure S7). These results point to the significant roles of these TFs in the regulation of leaf senescence in *M. truncatula* and warrant their detailed characterization through reverse genetic approaches in the future. The results also highlight the feasibility of using this transient expression system for the rapid screening of senescence-promoting TFs/genes.

## 4. Discussion

Forage yield and quality are negatively affected by the induction of age-dependent and stress-induced senescence. Delaying leaf senescence in forage legumes may therefore be a means of improving forage biomass production and quality, as well as to increase their resilience to certain kinds of stress. In this study, we employed physiological, transcriptomic and heterologous gene expression approaches to explore the molecular bases of leaf senescence and identify key transcription factors involved in the regulation of age-dependent and dark-induced senescence in *M. truncatula*. We found both similarities and differences in the genes that are engaged in *M. truncatula* during age-dependent and dark-induced senescence, which are discussed below.

Leaf yellowing is considered a hallmark of age-dependent leaf senescence that reflects a gradual decrease in anabolic activities and an increase in catabolic activities such as chlorophyll breakdown, which can be accelerated by stressful growth conditions including extended dark treatment, nutrient deprivation and drought stress [4,26,45]. Through the visual observation of leaf yellowing and measurement of chlorophyll levels in M3 leaves of *M. truncatula*, we recorded the gradual onset of senescence during leaf aging in contrast to rapid senescence in response to dark (Figure 1 and Figure 4). Consistent with these observations, the expressions of *RBCS*, *NYE1* and *PaO* were gradually altered during age-dependent senescence compared to dark-induced senescence where a drastic change in their expressions was recorded in dark-treated M3 leaves (Figure 1C and Figure 4C). Furthermore, transcriptomic data associated with age-dependent leaf senescence showed that M3 leaves retained some expression of PhAGs until the last day of sampling (42 DAS) likely because senescence did not occur synchronously in all cells, but rather was staged from marginal leaf cells inward. Thus, the transcript levels of several PhAGs decreased with leaf age as more cells entered senescence (Supplementary Dataset S2, S3 and S4). On the other hand, the dark treatment repressed a large number of PhAGs within 24 h and the transcript levels of these genes continued to decline in the following days. Similarly, we also observed a gradual reduction in the expression of genes associated with cell wall biogenesis and cytoskeleton during age-dependent senescence but a very rapid reduction in such genes during dark-induced senescence (Supplementary Dataset S5, S6 and S7). These findings are consistent with transcriptomic studies performed in other plant species in which the expression of genes associated with photosynthesis, cell wall biogenesis and the cytoskeleton were reduced during age-dependent and dark-induced leaf senescence [11,13,17,20,26,46,47].

The identification of co-expressed gene clusters/profiles can help associate genes with specific biological processes [34]. Using the Short Time-Series Expression Miner (STEM) program [34], we identified five distinct expression profiles/clusters for each upregulated and downregulated DEG in age-dependent senescence, four significant expression profiles for upregulated genes and six significant expression profiles for downregulated genes in dark-induced senescence (Figure 2C and Figure 5C and Supplementary Dataset S3 and S6). The GO-term enrichment analysis of the downregulated profiles identified several growth-related biological processes including photosynthesis, cell biogenesis (cellulose, hemicellulose and pectin) and phenylpropanoid pathways as commonly downregulated processes in *M. truncatula* during age-dependent and dark-induced senescence (Figure 7 and Supplementary Figures S3, S5 and Dataset S3, S4, S6–S8), consistent with previous observations in other plant species [11,13,17,20,26,47]. Genes related to mitochondrial functioning were strongly downregulated in response to dark treatment (Supplementary Dataset S3, S4, S6 and S7). Similar results were obtained in *A. thaliana* plants exposed to extended darkness, which exhibited the repression of genes associated with the mitochondrial electron transport chain, TCA cycle, and co-factor and vitamin metabolism [26]. In contrast, very little reduction in gene expression for mitochondrial functions was observed during age-dependent senescence, which is consistent with earlier reports that leaf mitochondria remain intact and active throughout senescence presumably to support catabolism and the remobilization of nutrients to other organs during age-dependent leaf senescence [48].

The analysis of cohorts of genes induced during senescence revealed that the activation of cell wall degradation and modification (cellulose, hemicellulose and pectin) followed the decline of cell biogenesis pathways, as found in other species [11,13,49,50]. Moreover, some genes related to cell wall modification such as expansins were specifically induced during dark treatment. The induction of expansins occurs during shading, heat and waterlogging stress to induce the leaf hyponastic response and is considered an important survival strategy under these stresses [51,52,53].

Studies in different plant species have shown that the expression of genes involved in lipid biosynthesis decrease and those involved in fatty acids turnover increase with age and during leaf senescence [54,55,56]. Consistent with these observations, the transcript levels of several genes related to lipid biosynthesis declined and those involved in lipid degradation increased during both age-dependent and dark-induced senescence in *Medicago*. However, a significantly higher number of genes related to lipid degradation were found amongst the upregulated genes of dark-induced senescence, possibly for the reason proposed above. These genes encoded enzymes involved in the degradation of phosopholipids, triglycerides and beta-oxidation (Supplementary Dataset S3, S4, S6 and S7).

Previous studies have shown that the levels of aromatic amino acids (Phe, Trp and Tyr), products of the shikimate pathway, and branched-chain amino acids (Val, Leu and Ile) increase during age-dependent and dark-induced leaf senescence [26,57,58,59,60,61]. Consistent with these studies, our upregulated profiles associated with both age-dependent and dark-induced senescence shared genes related to aromatic and branched-chain amino acid metabolism. Again, the induction of these genes was greater during dark-induced senescence (Supplementary Dataset S3, S4, S6 and S7). Aromatic amino acids serve as precursors for the synthesis of protective secondary metabolites such as flavonoids, whereas branched-chain amino acids can serve as the alternative respiratory substrates during leaf senescence [26,57,58,59,60,61]. Interestingly, several genes involved in flavonoid biosynthesis were also induced during age-dependent and dark-induced senescence (Supplementary Dataset S3, S4, S6 and S7). Plants activate several protein degradation pathways to ensure the efficient recycling and transport of nitrogen compounds from senescing leaves to other organs [46,62,63]. Consistent with this, we identified several genes for protein degradation enzymes/pathways common to both types of senescence and others specific to either age-dependent or dark-induced senescence. Fifty-six of the common genes encoded protein degradation enzymes such as subtilases, cysteine proteases, serine proteases, metalloproteases and the 26S proteasome pathway, including E2 conjugases and E3 ligases (Supplementary Dataset S3, S4, S6 and S7). Approximately 67 genes from similar protein degrading enzyme systems were upregulated in age-dependent senescence only. However, a much larger number (287 genes) were only found in the upregulated profiles of dark treated samples, encoding not only the aforementioned proteolytic enzymes, but also aspartate proteases (Supplementary Dataset S3, S4, S6 and S7). Interestingly, genes involved in autophagy were predominantly activated during dark-induced senescence compared to age-dependent senescence (Supplementary Dataset S3, S4, S6 and S7). Autophagy is predominantly required for the bulk recycling of macromolecular structures and organelles under age-dependent and stress-induced senescence and is normally associated with suppressed leaf senescence. However, once senescence is initiated, autophagy hastens this process [64]. In contrast, the 26S proteasome pathway was implicated in promoting the onset of age-dependent leaf senescence due to its ability to degrade specific regulatory proteins [64].

Reflecting and probably driving the stronger transcriptional response during dark-induced senescence compared to age-dependent senescence, we found a larger number of DE TFs and TF families associated with dark-induced senescence (Figure 4 and Figure 8). We identified at least 60 TF families associated with dark-induced leaf senescence compared to 48 TF families linked to age-dependent senescence (Figure 4 and Figure 8). This may simply reflect the stronger and/or more synchronous cellular induction of senescence by extended dark treatment and/or it may point to distinct transcriptional programs involved in dark-induced and age-dependent senescence. The major TF families identified during both age-dependent and dark-induced senescence included AP2/ERF, bHLH, bZIP, C2H2, GRAS, MYB/MYB-related, NAC, WRKY, C2C2-Dof, HSF and MADS-box (MIKC and M-type), with more family members being differentially expressed during dark-induced senescence (Figure 3 and Figure 6). Transcriptomic studies performed in different plant species have also implicated these TF families in leaf senescence [11,13,17,19,20,23,47,65]. In contrast to most TF families whose members exhibited both induction and reduction during senescence, members of the MADS-box (M-type) TF family were only induced during age-dependent and dark-induced senescence, suggesting a unique role for MADS-box (M-type) TFs during leaf senescence. Although a few members of the M-Type MADS subfamily have been characterized in floral development, their roles in the regulation of leaf senescence have not been studied [66,67,68]. Interestingly, members of the MICK-Type MADS subfamily have been shown to modulate leaf and petal senescence in *Arabidopsis* [69]. We also examined the frequency and types of TFs present in each distinct expression profile identified using STEM analysis. We found that downregulated expression profiles were mainly enriched in TF families normally associated with growth and photosynthesis such AP2/ERF, bHLH, GATA, GARP-G2 like, MYBs (Table 1 and Table 2). In *Arabidopsis*, TFs from GATA, MYB and G2-like families were implicated in chloroplast and chlorophyll biogenesis [70,71,72]. On the other hand, upregulated expression profiles were enriched with TFs implicated in the regulation of stress responses, including leaf senescence. These included TFs from families such as bZIP, bHLH, MYB, NAC and WRKY (Table 1 and Table 2). Several TFs belonging to these TF families have been shown to promote leaf senescence in different plant species [18,21,22,24,73,74,75,76,77,78,79,80].

We also compared our age-dependent and dark-induced transcriptomic data with *Arabidopsis* developmental time-series transcriptomic data reported by Breeze et al. [11] and dark-induced senescence by Law *et al*. [26]. Approximately 15.7% (1563) of DEGs were shared between the age-dependent transcriptomic datasets of *M. truncatula* and *A. thaliana*, and 20.2% (2102) of DEGs were shared between *M. truncatula* and *A. thaliana* dark-induced senescence transcriptome datasets (Figure 8A,D), indicating substantial conservation as well as divergence between the senescence pathways of these two species. These results are consistent with similar transcriptomic comparisons among other plants species [17,19,65].

Recently, dark-induced and salinity-induced senescence transcriptomes were compared in *M. truncatula*, using a detached leaf system [81]. Unlike the intact plant system employed in our study, detached leaves add layers of complexity associated with physical damage to leaves and the severance of long-distance transport and signaling pathways connecting leaves to the rest of the plant, which complicates the interpretation of transcriptional responses to stress treatments. Not surprisingly, our gene expression profiles of dark-treated intact plants substantially differed from those of dark-treated detached leaves, exhibiting an overlap of only 30% (2671) of genes induced and approximately 35% (3705) of genes repressed by dark treatment (Supplemental Figure S8).

Although transcriptomic studies in different plant species have identified many TFs potentially involved in the regulation of leaf senescence, the functional roles of most of these remain unknown [11,17,26,49,65]. The generation and screening of mutants and transgenic approaches to ascertain gene function are time and resource consuming processes. In this study, we employed a transient expression system using tobacco for the rapid screening of selected SAG-TFs for their ability to trigger senescence. We validated this system by transiently overexpressing two known senescence-promoting TFs, ORE1/ANAC092 and AtNAP from *A. thaliana* (Figure 9B) [15,36,70]. We then transiently overexpressed 26 core TFs that were induced during age-dependent or dark-induced senescence, or both, and found that 13 of these triggered leaf senescence/cell death symptoms when expressed in tobacco leaves. These included putative orthologs of AtNAP and ANAC072/RD26, Medtr4g081870 and Medtr8g059170, respectively. The overexpression of AtNAP and ANAC072/RD26 in *Arabidopsis* is known to induce leaf senescence [36,82]. Other TFs that promoted senescence in tobacco leaves belonged to the MYB, NAC, C2H2, bZIP, HD-Zip and WKRY families (Figure 9C and Supplementary Figure S7 and Table S2). Previously, the use of the transient gene expression system in tobacco leaves enabled the identification of several TFs that promote suberin biosynthesis in *A. thaliana* [44], giving us confidence that the TFs identified here have bone fide roles in plant senescence.

## 5. Conclusions

Age-dependent and dark-induced senescence in *M. truncatula* were found to involve both common and distinct genes and processes, including the transcriptional regulators of these processes. Among the 26 transcription factors induced during senescence in *M. truncatula* that were subsequently expressed in tobacco leaves, at least 13 induced senescence in those leaves. Therefore, these are promising targets for future work to determine their specific roles in senescence and for mutational approaches to alter leaf senescence in forage species with the aim of increasing forage production and quality.

## Figures and Tables

**Table 1:** Transcription factor families with distinct expression kinetics during age-dependent senescence identified via STEM analysis.

Families	0 Profile	6 Profile	7 Profile	10 Profile	18 Profile	31 Profile	34 Profile	35 Profile	38 Profile	39 Profile
AP2/ERF-ERF	4	6	1	-	1	5	-	3	8	2
B3-ARF	-	1	-	-	-	-	-	-	-	-
bHLH	1	5	-	1	1	3	-	2	1	2
bZIP	-	1	-	3	1	2	-	4	3	-
C2C2-CO-like	-	2	-	1	-	-	-	-	-	-
C2C2-Dof	-	2	-	-	1	1	-	1	1	-
C2C2-GATA	1	1	-	2	-	-	-	-	1	1
C2C2-YABBY	-	1	-	-	-	-	-	-	-	-
C2H2	3	-	1	1	-	5	1	-	1	1
C3H	2	2	-	-	-	1	-	-	-	-
E2F-DP	-	-	-	-	-	1	-	-	-	-
FAR1	-	-	-	-	-	1	-	1	-	-
GARP-G2-like	1	3	-	-	-	1	-	1	1	-
GRAS	-	-	-	2	-	2	-	4	2	-
GRF	-	-	-	1	-	-	-	-	-	-
HB-BELL	-	-	-	-	-	-	-	1	-	-
HB-HD-ZIP	-	3	1	2	-	1	-	2	-	-
HB-KNOX	-	-	-	-	-	-	-	1	-	-
HB-other	-	2	-	-	-	-	-	-	-	-
HSF	-	-	2	-	-	-	-	1	-	1
LBD	1	-	-	-	-	-	-	2	1	1
LIM	-	-	-	1	-	-	-	-	-	-
MADS-MIKC	1	-	-	-	-	-	-	5	-	-
MADS-M-type	-	-	-	-	-	2	-	1	-	-
MYB	10	5	2	-	-	2	1	3	2	1
MYB-related	-	2	-	2	-	1	-	1	-	2
NAC	4	5	-	1	-	3	-	3	2	-
NF-YA	-	-	-	-	-	1	-	1	1	-
NF-YB	-	1	-	-	-	-	-	2	-	-
OFP	1	1	-	3	-	2	-	1	-	-
PLATZ	-	2	-	-	-	-	-	-	-	-
SBP	1	1	-	-	-	-	-	-	-	-
SRS	-	-	-	-	-	-	-	-	-	1
TCP	-	1	-	-	-	-	-	1	-	-
Tify	-	-	3	-	-	-	-	1	-	-
Trihelix	1	-	-	-	-	1	-	2	-	-
TUB	-	-	-	1	-	-	-	-	-	-
ULT	-	-	-	-	-	-	-	-	-	1
Whirly	-	-	-	-	-	-	-	-	-	1
WRKY	8	5	-	-	3	1	-	9	1	-
zf-HD	-	1	-	-	-	-	1	1	-	1

**Table 2:** Transcription factors’ families with distinct expression kinetics during dark-induced senescence, identified via STEM analysis.

Families	0 Profile	1 Profile	2 Profile	3 Profile	6 Profile	18 Profile	23 Profile	35 Profile	37 Profile	39 Profile
Alfin-like	-	-	-	-	1	-	-	-	-	-
AP2/ERF-AP2	2	-	-	1	2	-	-	-	1	-
AP2/ERF-ERF	10	3	5	1	14	2	-	9	2	9
AP2/ERF-RAV	-	-	-	-	-	-	-	-	-	1
B3	1	-	1	-	1	1	1	3	2	1
B3-ARF	2	1	4	-	1	-	-	2	-	-
BBR-BPC	-	-	-	-	-	-	-	1	-	-
bHLH	9	3	5	2	13	-	-	11	1	5
bZIP	3	2	1	3	2	-	-	9	4	6
C2C2-CO-like	-	-	-	-	3	-	2	-	1	-
C2C2-Dof	2	-	1	1	2	-	6	5	-	-
C2C2-GATA	3	-	-	1	3	-	-	1	-	2
C2C2-LSD	-	-	-	-	-	-	-	-	-	1
C2C2-YABBY	1	-	1	-	1	-	-	-	-	-
C2H2	5	2	3	2	6	-	1	7	-	6
C3H	4	-	-	-	3	-	-	1	-	2
CAMTA	1	-	-	-	-	-	-	2	1	-
CPP	-	-	-	1	-	-	-	-	-	-
DBB	1	-	-	1	-	-	1	-	-	1
DBP	-	1	-	-	-	-	-	-	-	-
E2F-DP	-	-	-	-	1	-	-	1	-	1
EIL	-	-	-	-	-	-	-	1	-	-
FAR1	-	-	-	-	-	-	-	2	-	3
GARP-ARR-B	-	-	-	1	-	1	-	1	-	2
GARP-G2-like	1	1	1	2	2	2	1	1	1	3
GeBP	1	-	-	-	1	-	-	1	-	-
GRAS	10	3	1	-	4	3	-	6	-	2
GRF	-	-	-	-	1	-	-	-	-	1
HB-BELL	-	-	1	-	-	-	-	2	1	1
HB-HD-ZIP	4	3	-	1	5	1	2	4	-	2
HB-KNOX	1	-	-	-	-	-	-	-	1	-
HB-other	-	-	-	-	-	-	-	2	1	1
HB-WOX	1	-	-	-	1	-	-	-	-	-
HRT	-	-	-	-	1	-	-	-	-	-
HSF	1	1	1	1	-	-	-	3	2	3
LBD	-	-	1	1	3	-	1	4	1	2
LIM	-	-	1	-	-	-	-	-	-	-
MADS-MIKC	-	2	2	1	1	1	-	3	-	1
MADS-M-type	-	-	-	-	-	-	-	6	1	1
MYB	10	-	5	-	9	1	2	11	2	7
MYB-related	6	2	1	-	1	1	3	8	1	4
NAC	3	1	2	2	5	-	1	21	-	5
NF-X1	-	-	-	-	-	-	-	1	-	1
NF-YA	-	-	-	-	1	-	-	3	-	-
NF-YB	1	1	-	-	-	-	-	3	-	-
NF-YC	-	-	1	-	-	-	-	1	-	1
OFP	1	1	-	1	3	-	-	2	-	1
PLATZ	-	-	-	-	2	-	-	1	-	2
RWP-RK	-	-	-	-	2	-	-	-	-	1
S1Fa-like	-	-	-	-	1	-	-	-	-	-
SBP	1	-	-	-	4	1	-	-	1	1
SRS	-	-	1	-	-	-	-	-	-	-
STAT	1	-	-	-	-	-	-	-	-	-
TCP	2	1	1	-	3	-	-	2	-	1
Tify	1	-	1	-	-	-	1	4	-	-
Trihelix	-	-	-	1	-	-	-	4	-	2
TUB	-	-	-	-	2	-	-	-	-	-
ULT	-	1	-	-	-	-	-	-	-	-
WRKY	2	1	1	-	12	6	2	6	2	2
zf-HD	1	-	2	1	1	1	-	1	-	-

## Data Availability

Raw RNA-seq data corresponding to age-dependent and dark-induced senescence could be accessed at https://www.ncbi.nlm.nih.gov/sra/PRJNA832418.

## Supplementary Materials

The following supporting information can be downloaded at: https://www.mdpi.com/article/10.3390/cells11091570/s1, Figure S1: Chlorophyll analysis in main metameric leaves of A17; Figure S2: STEM analysis of differentially expressed genes identified during age-dependent senescence; Figure S3: GO-term enrichment analysis of discrete DE/SAG expression profiles during development senescence; Figure S4: STEM analysis of differentially expressed genes identified during dark-induced senescence; Figure S5. GO-term enrichment analysis of discrete DE/SAG expression profiles during dark treatment; Figure S6: Comparison of GO terms specifically associated with age-dependent and dark-induced senescence; Figure S7: Transient overexpression of selected SA-TFs in tobacco leaves; Figure S8: Comparison of transcriptomic data from dark-treated intact plants and dark-treated detached leaves in *M. truncatula*; Figure S9. Confirmation of RNA-seq data by comparing the expression patterns of the *RBCS, PaO* and *NYE1* analyzed by quantitative RT-PCR with the RNA-seq FPKM values; Table S1: List of primer sequences used in the study; Table S2: List of senescence-induced transcription factors transiently overexpressed in tobacco leaves; Dataset S1: FPKM values of expressed genes during age-dependent and dark-induced senescence; Dataset S2: Log2 fold change values of DEGs during age-dependent senescence; Dataset S3: STEM profiles with Mapman annotations of DEGs during age-dependent senescence; Dataset S4: GO terms based on STEM profiles of DEGs during age-dependent senescence; Dataset S5: Log2 fold changes values of DEGs during dark-induced senescence; Dataset S6: STEM profiles with Mapman annotations of DEGs during dark-induced senescence; Dataset S7: GO terms based on STEM clusters of DEGs during dark-induced senescence; and Dataset S8: Comparison of age-dependent and dark-induced senescence transcriptomic profiles.

## References

[1] Yang J., Udvardi M. (2018). Senescence and Nitrogen Use Efficiency in Perennial Grasses for Forage and Biofuel Production. J. Exp. Bot..

[2] Avila-Ospina L., Moison M., Yoshimoto K., Masclaux-Daubresse C. (2014). Autophagy, Plant Senescence, and Nutrient Recycling. J. Exp. Bot..

[3] Gregersen P.L., Culetic A., Boschian L., Krupinska K. (2013). Plant Senescence and Crop Productivity. Plant Mol. Biol..

[4] Lim P.O., Kim H.J., Nam H.G. (2007). Leaf Senescence. Annu. Rev. Plant Biol..

[5] Clifton-Brown J.C., Lewandowski I. (2002). Screening Miscanthus Genotypes in Field Trials to Optimise Biomass Yield and Quality in Southern Germany. Eur. J. Agron..

[6] Rivero R.M., Kojima M., Gepstein A., Sakakibara H., Mittler R., Gepstein S., Blumwald E. (2007). Delayed Leaf Senescence Induces Extreme Drought Tolerance in a Flowering Plant. Proc. Natl. Acad. Sci. USA.

[7] Sakuraba Y., Kim Y.S., Han S.H., Lee B.D., Paek N.C. (2015). The Arabidopsis Transcription Factor NAC016 Promotes Drought Stress Responses by Repressing AREB1 Transcription through a Trifurcate Feed-Forward Regulatory Loop Involving NAP. Plant Cell.

[8] Sakuraba Y., Piao W., Lim J.H., Han S.H., Kim Y.S., An G., Paek N.C. (2015). Rice ONAC106 Inhibits Leaf Senescence and Increases Salt Tolerance and Tiller Angle. Plant Cell Physiol..

[9] Thomas H., Howarth C.J. (2000). Five Ways to Stay Green. J. Exp. Bot..

[10] Yu G., Xie Z., Zhang J., Lei S., Lin W., Xu B., Huang B. (2021). NOL-Mediated Functional Stay-Green Traits in Perennial Ryegrass (*Lolium perenne* L.) Involving Multifaceted Molecular Factors and Metabolic Pathways Regulating Leaf Senescence. Plant J..

[11] Breeze E., Harrison E., McHattie S., Hughes L., Hickman R., Hill C., Kiddle S., Kim Y.-s., Penfold C.A., Jenkins D. (2011). High-Resolution Temporal Profiling of Transcripts during Arabidopsis Leaf Senescence Reveals a Distinct Chronology of Processes and Regulation. Plant Cell.

[12] Kim J., Woo H.R.R., Nam H.G.G. (2016). Toward Systems Understanding of Leaf Senescence: An Integrated Multi-Omics Perspective on Leaf Senescence Research. Mol. Plant.

[13] Buchanan-Wollaston V., Page T., Harrison E., Breeze E., Lim P.O., Nam H.G., Lin J.-F., Wu S.-H., Swidzinski J., Ishizaki K. (2005). Comparative Transcriptome Analysis Reveals Significant Differences in Gene Expression and Signalling Pathways between Developmental and Dark/Starvation-Induced Senescence in Arabidopsis. Plant J..

[14] de Michele R., Formentin E., Todesco M., Toppo S., Carimi F., Zottini M., Barizza E., Ferrarini A., Delledonne M., Fontana P. (2009). Transcriptome Analysis of Medicago Truncatula Leaf Senescence: Similarities and Differences in Metabolic and Transcriptional Regulations as Compared with Arabidopsis, Nodule Senescence and Nitric Oxide Signalling. New Phytol..

[15] Balazadeh S., Siddiqui H., Allu A.D., Matallana-Ramirez L.P., Caldana C., Mehrnia M., Zanor M.-I., Köhler B., Mueller-Roeber B. (2010). A Gene Regulatory Network Controlled by the NAC Transcription Factor ANAC092/AtNAC2/ORE1 during Salt-Promoted Senescence. Plant J..

[16] Besseau S., Li J., Palva E.T. (2012). WRKY54 and WRKY70 Co-Operate as Negative Regulators of Leaf Senescence in Arabidopsis Thaliana. J. Exp. Bot..

[17] Sekhon R.S., Childs K.L., Santoro N., Foster C.E., Buell C.R., de Leon N., Kaeppler S.M. (2012). Transcriptional and Metabolic Analysis of Senescence Induced by Preventing Pollination in Maize. Plant Physiol..

[18] Song Y., Yang C., Gao S., Zhang W., Li L., Kuai B. (2014). Age-Triggered and Dark-Induced Leaf Senescence Require the BHLH Transcription Factors PIF3, 4, and 5. Mol. Plant.

[19] Lin M., Pang C., Fan S., Song M., Wei H., Yu S. (2015). Global Analysis of the Gossypium Hirsutum L. Transcriptome during Leaf Senescence by RNA-Seq. BMC Plant Biol..

[20] Brown A.V., Hudson K.A. (2015). Developmental Profiling of Gene Expression in Soybean Trifoliate Leaves and Cotyledons. BMC Plant Biol..

[21] Gao S., Gao J., Zhu X., Song Y., Li Z., Ren G., Zhou X., Kuai B. (2016). ABF2, ABF3, and ABF4 Promote ABA-Mediated Chlorophyll Degradation and Leaf Senescence by Transcriptional Activation of Chlorophyll Catabolic Genes and Senescence-Associated Genes in Arabidopsis. Mol. Plant.

[22] Mahmood K., El-Kereamy A., Kim S.H., Nambara E., Rothstein S.J. (2016). ANAC032 Positively Regulates Age-Dependent and Stress-Induced Senescence in Arabidopsis Thaliana. Plant Cell Physiol..

[23] Wu X.Y., Hu W.J., Luo H., Xia Y., Zhao Y., Wang L.D., Zhang L.M., Luo J.C., Jing H.C. (2016). Transcriptome Profiling of Developmental Leaf Senescence in Sorghum (Sorghum Bicolor). Plant Mol. Biol..

[24] Mao C., Lu S., Lv B., Zhang B., Shen J., He J., Luo L., Xi D., Chen X., Ming F. (2017). A Rice Nac Transcription Factor Promotes Leaf Senescence via ABA Biosynthesis. Plant Physiol..

[25] Sakuraba Y., Kim D., Han S.H., Kim S.H., Piao W., Yanagisawa S., An G., Paek N.C. (2020). Multilayered Regulation of Membrane-Bound ONAC054 Is Essential for Abscisic Acid-Induced Leaf Senescence in Rice. Plant Cell.

[26] Law S.R., Chrobok D., Juvany M., Delhomme N., Lindén P., Brouwer B., Ahad A., Moritz T., Jansson B.S., Gardeström P. (2018). Darkened Leaves Use Different Metabolic Strategies for Senescence and Survival. Plant Physiol..

[27] Bucciarelli B., Hanan J., Palmquist D., Vance C.P. (2006). A Standardized Method for Analysis of Medicago truncatula Phenotypic Development. Plant Physiol..

[28] Sestak Z., Catsky J., Jarvis P. (1971). Plant Photosynthetic Production. Manual of Methods.

[29] Chomczynski P., Mackey K. (1995). Short technical reports. Modification of the TRI reagent procedure for isolation of RNA from polysaccharide-and proteoglycan-rich sources. Biotechniques.

[30] Kakar K., Wandrey M., Czechowski T., Gaertner T., Scheible W.R., Stitt M., Udvardi M.K. (2008). A community resource for high-throughput quantitative RT-PCR analysis of transcription factor gene expression in Medicago truncatula. Plant Methods.

[31] Ernst J., Bar-Joseph Z. (2006). STEM: A Tool for the Analysis of Short Time Series Gene Expression Data. BMC Bioinform..

[32] Tian F., Yang D.C., Meng Y.Q., Jin J., Gao G. (2020). PlantRegMap: Charting Functional Regulatory Maps in Plants. Nucleic Acids Res..

[33] Supek F., Bošnjak M., Škunca N., Šmuc T. (2011). Revigo Summarizes and Visualizes Long Lists of Gene Ontology Terms. PLoS ONE.

[34] Sun L., Dong S., Ge Y., Fonseca J.P., Robinson Z.T., Mysore K.S., Mehta P. (2019). DiVenn: An interactive and integrated web-based visualization tool for comparing gene lists. Front. Genet..

[35] Gibson D.G., Young L., Chuang R.Y., Venter J.C., Hutchison C.A., Smith H.O. (2009). Enzymatic assembly of DNA molecules up to several hundred kilobases. Nat. Methods.

[36] Guo Y., Gan S. (2006). AtNAP, a NAC Family Transcription Factor, Has an Important Role in Leaf Senescence. Plant J..

[37] Jiang Y., Liang G., Yang S., Yu D. (2014). Arabidopsis WRKY57 Functions as a Node of Convergence for Jasmonic Acid- and Auxin-Mediated Signaling in Jasmonic Acid-Induced Leaf Senescence. Plant Cell.

[38] Guo P., Li Z., Huang P., Li B., Fang S., Chu J., Guo H. (2017). A Tripartite Amplification Loop Involving the Transcription Factor WRKY75, Salicylic Acid, and Reactive Oxygen Species Accelerates Leaf Senescence. Plant Cell.

[39] Xiao S., Gao W., Chen Q.F., Chan S.W., Zheng S.X., Ma J., Wang M., Welti R., Chye M.L. (2010). Overexpression of Arabidopsis Acyl-CoA Binding Protein ACBP3 Promotes Starvation-Induced and Age-Dependent Leaf Senescence. Plant Cell.

[40] Yang Y., Li R., Qi M. (2000). In Vivo Analysis of Plant Promoters and Transcription Factors by Agroinfiltration of Tobacco Leaves. Plant J..

[41] Ueda H., Yamaguchi Y., Sano H. (2006). Direct Interaction between the Tobacco Mosaic Virus Helicase Domain and the ATP-Bound Resistance Protein, N Factor during the Hypersensitive Response in Tobacco Plants. Plant Mol. Biol..

[42] Park S.Y., Yu J.W., Park J.S., Li J., Yoo S.C., Lee N.Y., Lee S.K., Jeong S.W., Hak S.S., Koh H.J. (2007). The Senescence-Induced Staygreen Protein Regulates Chlorophyll Degradation. Plant Cell.

[43] Lim S.H., Sohn S.H., Kim D.H., Kim J.K., Lee J.Y., Kim Y.M., Ha S.H. (2012). Use of an Anthocyanin Production Phenotype as a Visible Selection Marker System in Transgenic Tobacco Plant. Plant Biotechnol. Rep..

[44] Kosma D.K., Murmu J., Razeq F.M., Santos P., Bourgault R., Molina I., Rowland O. (2014). AtMYB41 Activates Ectopic Suberin Synthesis and Assembly in Multiple Plant Species and Cell Types. Plant J..

[45] Peng M., Hannam C., Gu H., Bi Y.M., Rothstein S.J. (2007). A mutation in NLA, which encodes a RING-type ubiquitin ligase, disrupts the adaptability of Arabidopsis to nitrogen limitation. Plant J..

[46] Keech O., Pesquet E., Gutierrez L., Ahad A., Bellini C., Smith S.M., Gardeström P. (2010). Leaf Senescence Is Accompanied by an Early Disruption of the Microtubule Network in Arabidopsis. Plant Physiol..

[47] Sobieszczuk-Nowicka E., Wrzesiński T., Bagniewska-Zadworna A., Kubala S., Rucińska-Sobkowiak R., Polcyn W., Misztal L., Mattoo A.K. (2018). Physio-Genetic Dissection of Dark-Induced Leaf Senescence and Timing Its Reversal in Barley. Plant Physiol..

[48] Chrobok D., Law S.R., Brouwer B., Lindén P., Ziolkowska A., Liebsch D., Narsai R., Szal B., Moritz T., Rouhier N. (2016). Dissecting the Metabolic Role of Mitochondria during Developmental Leaf Senescence. Plant Physiol..

[49] Sekhon R.S., Saski C., Kumar R., Flinn B.S., Luo F., Beissinger T.M., Kaeppler S.M. (2019). Integrated genome-scale analysis identifies novel genes and networks underlying senescence in maize. Plant Cell.

[50] Borniego M.L., Molina M.C., Guiamét J.J., Martinez D.E. (2020). Physiological and proteomic changes in the apoplast accompany leaf senescence in Arabidopsis. Front. Plant Sci..

[51] Sasidharan R., Chinnappa C.C., Voesenek L.A.C.J., Pierik R. (2008). The Regulation of Cell Wall Extensibility during Shade Avoidance: A Study Using Two Contrasting Ecotypes of Stellaria Longipes. Plant Physiol..

[52] Rauf M., Arif M., Fisahn J., Xue G.P., Balazadeh S., Mueller-Roeber B. (2013). NAC Transcription Factor SPEEDY HYPONASTIC GROWTH Regulates Flooding-Induced Leaf Movement in Arabidopsis. Plant Cell.

[53] Marowa P., Ding A., Kong Y. (2016). Expansins: Roles in Plant Growth and Potential Applications in Crop Improvement. Plant Cell Rep..

[54] Pollard M., Ohlrogge J. (1999). Testing Models of Fatty Acid Transfer and Lipid Synthesis in Spinach Leaf Using In Vivo Oxygen-18 Labeling. Plant Physiol..

[55] Yang Z., Ohlrogge J.B. (2009). Turnover of Fatty Acids during Natural Senescence of Arabidopsis, Brachypodium, and Switchgrass and in Arabidopsis β-Oxidation Mutants. Plant Physiol..

[56] Troncoso-Ponce M.A., Cao X., Yang Z., Ohlrogge J.B. (2013). Lipid Turnover during Senescence. Plant Sci..

[57] Diaz C., Purdy S., Christ A., Morot-Gaudry J.F., Wingler A., Masclaux-Daubresse C. (2005). Characterization of Markers to Determine the Extent and Variability of Leaf Senescence in Arabidopsis. A Metabolic Profiling Approach. Plant Physiol..

[58] Araújo W.L., Ishizaki K., Nunes-Nesi A., Larson T.R., Tohge T., Krahnert I., Witt S., Obata T., Schauer N., Graham I.A. (2010). Identification of the 2-Hydroxyglutarate and Isovaleryl-CoA Dehydrogenases as Alternative Electron Donors Linking Lysine Catabolism to the Electron Transport Chain of Arabidopsis Mitochondria. Plant Cell.

[59] Araújo W.L., Tohge T., Ishizaki K., Leaver C.J., Fernie A.R. (2011). Protein Degradation-an Alternative Respiratory Substrate for Stressed Plants. Trends Plant Sci..

[60] Watanabe M., Balazadeh S., Tohge T., Erban A., Giavalisco P., Kopka J., Mueller-Roeber B., Fernie A.R., Hoefgen R. (2013). Comprehensive Dissection of Spatiotemporal Metabolic Shifts in Primary, Secondary, and Lipid Metabolism during Developmental Senescence in Arabidopsis. Plant Physiol..

[61] Li W., Zhang H., Li X., Zhang F., Liu C., Du Y., Gao X., Zhang Z., Zhang X., Hou Z. (2017). Intergrative Metabolomic and Transcriptomic Analyses Unveil Nutrient Remobilization Events in Leaf Senescence of Tobacco. Sci. Rep..

[62] Liu J., Wu Y.H., Yang J.J., Liu Y.D., Shen F.F. (2008). Protein degradation and nitrogen remobilization during leaf senescence. J. Plant Biol..

[63] Xie Q., Michaeli S., Peled-Zehavi H., Galili G. (2015). Chloroplast degradation: One organelle, multiple degradation pathways. Trends Plant Sci..

[64] Wang H., Schippers J.H.M. (2019). The Role and Regulation of Autophagy and the Proteasome during Aging and Senescence in Plants. Genes.

[65] Borrill P., Harrington S.A., Simmonds J., Uauy C. (2019). Identification of Transcription Factors Regulating Senescence in Wheat through Gene Regulatory Network Modelling. Plant Physiol..

[66] Köhler C., Hennig L., Spillane C., Pien S., Gruissem W., Grossniklaus U. (2003). The Polycomb-Group Protein MEDEA Regulates Seed Development by Controlling Expression of the MADS-Box Gene PHERES1. Genes Dev..

[67] Walia H., Josefsson C., Dilkes B., Kirkbride R., Harada J., Comai L. (2009). Dosage-Dependent Deregulation of an AGAMOUS-LIKE Gene Cluster Contributes to Interspecific Incompatibility. Curr. Biol..

[68] Bemer M., Wolters-Arts M., Grossniklaus U., Angenenta G.C. (2008). The MADS Domain Protein DIANA Acts Together with AGAMOUS-LIKE80 to Specify the Central Cell in Arabidopsis Ovules. Plant Cell.

[69] Chen M.K., Hsu W.H., Lee P.F., Thiruvengadam M., Chen H.I., Yang C.H. (2011). The MADS Box Gene, FOREVER YOUNG FLOWER, Acts as a Repressor Controlling Floral Organ Senescence and Abscission in Arabidopsis. Plant J..

[70] Rauf M., Arif M., Dortay H., Matallana-Ramírez L.P., Waters M.T., Gil Nam H., Lim P.-O., Mueller-Roeber B., Balazadeh S. (2013). ORE1 Balances Leaf Senescence against Maintenance by Antagonizing G2-like-Mediated Transcription. EMBO Rep..

[71] Hudson D., Guevara D.R., Hand A.J., Xu Z., Hao L., Chen X., Zhu T., Bi Y.M., Rothstein S.J. (2013). Rice Cytokinin GATA Transcription Factor1 Regulates Chloroplast Development and Plant Architecture. Plant Physiol..

[72] Ampomah-Dwamena C., Thrimawithana A.H., Dejnoprat S., Lewis D., Espley R.V., Allan A.C. (2019). A Kiwifruit (Actinidia Deliciosa) R2R3-MYB Transcription Factor Modulates Chlorophyll and Carotenoid Accumulation. New Phytol..

[73] Zhang X., Ju H.W., Chung M.S., Huang P., Ahn S.J., Kim C.S. (2011). The R-R-Type MYB-like Transcription Factor, AtMYBL, Is Involved in Promoting Leaf Senescence and Modulates an Abiotic Stress Response in Arabidopsis. Plant Cell Physiol..

[74] Yang J., Worley E., Udvardi M. (2014). A NAP-AAO3 Regulatory Module Promotes Chlorophyll Degradation via Aba Biosynthesis in Arabidopsis Leaves. Plant Cell.

[75] Qi T., Wang J., Huang H., Liu B., Gao H., Liu Y., Song S., Xie D. (2015). Regulation of Jasmonate-Induced Leaf Senescence by Antagonism between BHLH Subgroup IIIe and IIId Factors in Arabidopsis. Plant Cell.

[76] Chen L., Xiang S., Chen Y., Li D., Yu D. (2017). Arabidopsis WRKY45 Interacts with the DELLA Protein RGL1 to Positively Regulate Age-Triggered Leaf Senescence. Mol. Plant.

[77] Uji Y., Akimitsu K., Gomi K. (2017). Identification of OsMYC2-Regulated Senescence-Associated Genes in Rice. Planta.

[78] Kim T., Kang K., Kim S.H., An G., Paek N.C. (2019). OsWRKY5 Promotes Rice Leaf Senescence via Senescence-Associated NAC and Abscisic Acid Biosynthesis Pathway. Int. J. Mol. Sci..

[79] Zhang J., Fengler K.A., van Hemert J.L., Gupta R., Mongar N., Sun J., Allen W.B., Wang Y., Weers B., Mo H. (2019). Identification and Characterization of a Novel Stay-Green QTL That Increases Yield in Maize. Plant Biotechnol. J..

[80] Kim J.H., Woo H.R., Kim J., Lim P.O., Lee I.C., Choi S.H., Hwang D., Nam H.G. (2009). Trifurcate Feed-Forward Regulation of Age-Dependent Cell Death Involving MiR164 in Arabidopsis. Science.

[81] Dong S., Sang L., Xie H., Chai M., Wang Z.Y. (2021). Comparative transcriptome analysis of salt stress-induced leaf senescence in Medicago truncatula. Front. Plant Sci..

[82] Li S., Gao J., Yao L., Ren G., Zhu X., Gao S., Kuai B. (2016). The role of ANAC072 in the regulation of chlorophyll degradation during age-and dark-induced leaf senescence. Plant Cell Rep..

