# Supplementary material for: Transcriptional Programs and Regulators Underlying Age-Dependent and Dark-Induced Senescence in Medicago truncatula
Source: Cells. 2022 May 6;11(9):1570. doi: 10.3390/cells11091570 (PMC9103780; doi:10.3390/cells11091570)
Supplement: Supplementary file 1 [file cells-11-01570-s001.zip › Revised Supplementary material_Mahmood et al., cells-1648109.pdf]

Supplementary Material

Transcriptional programs and regulators underlying age-dependent and dark-induced senescence in *Medicago truncatula*

Kashif Mahmood[1, 2], Ivone Torres-Jerez[1, 2], Nick Krom[1], Wei Liu[1, 3], Michael K. Udvardi [2, 4 *]

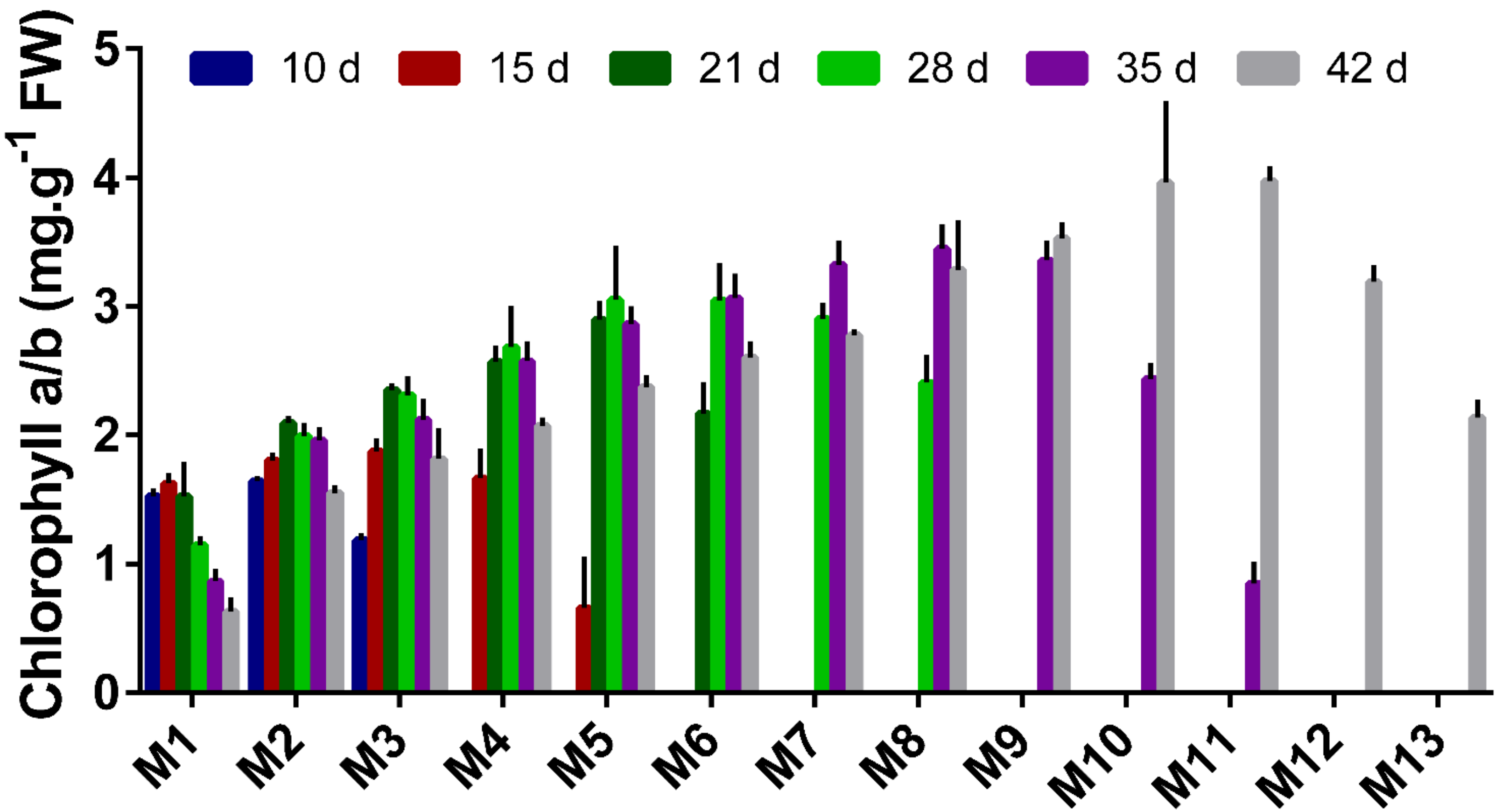

Figure S1. Chlorophyll analysis in main metameric leaves of A17. Seedlings of A17 plants were transplanted into soil one day after seed germination and grown for 42 days under long-day growth conditions. All the main metameric leaves were harvested from plants 10, 15, 21, 28, 35 and 42 DAS (pooled from six individual plants as one biological replicate). Chlorophyll was extracted using 80% acetone and absorbance of supernatants were measured spectrophotometrically at 645 nm and 663 nm. Data represent SD± mean values from four biological replicates and statistically analyzed using one-way ANOVA.

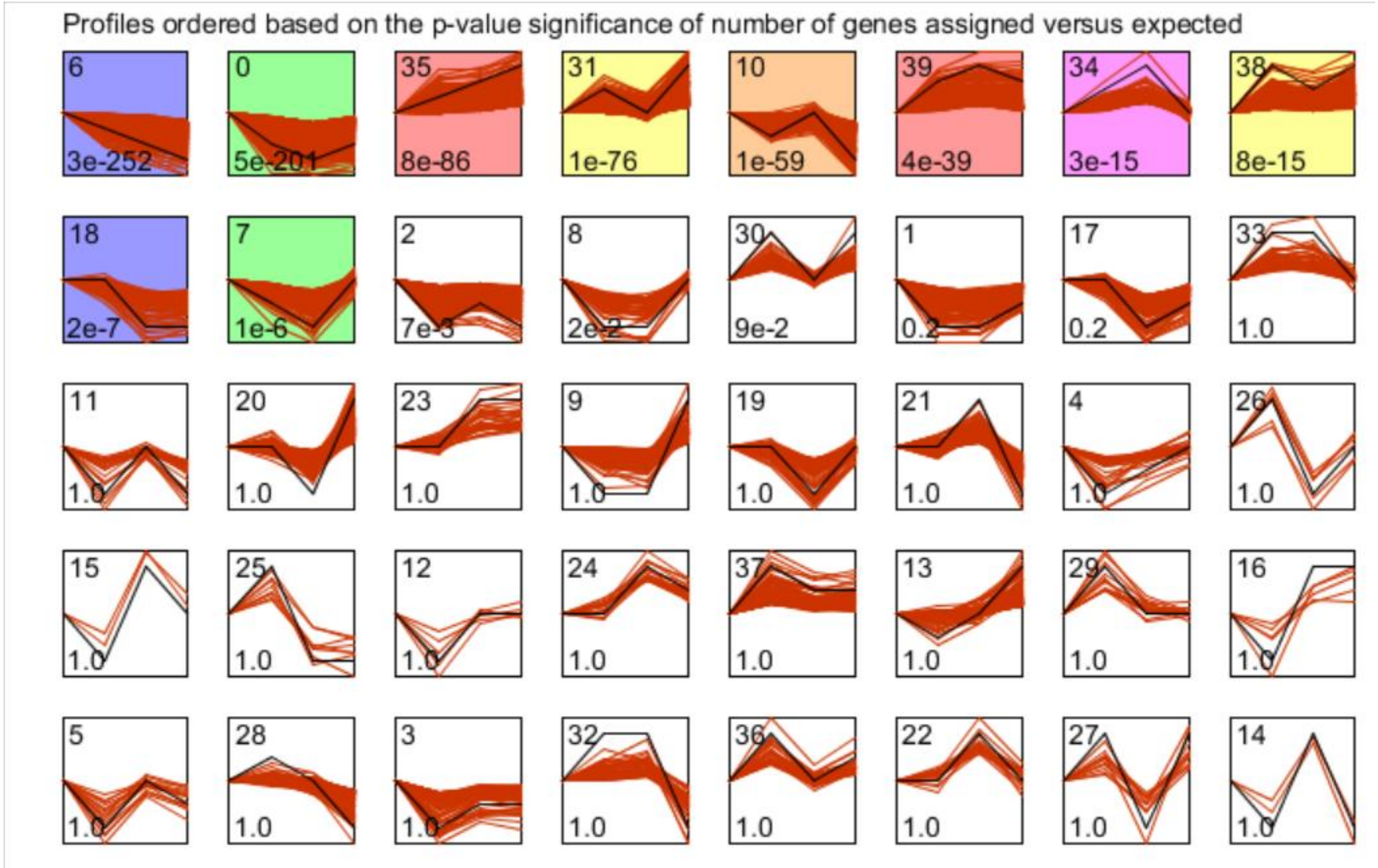

Figure S2. STEM analysis of differentially expressed genes identified during age-dependent senescence. Numbers in the top left panel represent gene profiles or clusters and the numbers in the left bottom represent the P values. Profiles with P values ≤ 0.05 are considered significant. Analysis was performed using default parameters.

Profile #	GO term	P value	Description
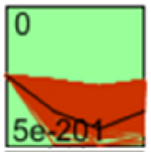 0 / 5e-201	GO:0006952	3.20E-10	Defense response
	GO:0007154	2.00E-07	Cell communication
	GO:0006950	1.10E-06	Response to Stress
	GO:0007154	2.40E-06	Signal transduction
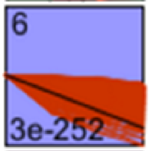 6 / 3e-252	GO:0071554	2.20E-11	Cell wall organization/biogenesis
	GO:0005975	4.80E-10	Carbohydrate metabolic process
	GO:0009698	1.30E-07	Phenylpropanoid metabolic process
	GO:0009808	1.40E-06	Lignin metabolic process
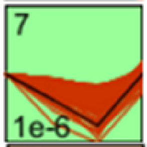 7 / 1e-6	GO:1901700	3.30E-05	Response to oxygen-containing compound
	GO:0009611	4.00E-04	Response to wounding
	GO:0009753	4.00E-04	Response to jasmonic acid
	GO:0001101	5.10E-04	Response to acid chemical
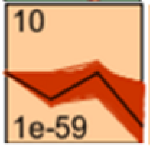 10 / 1e-59	GO:0015979	4.50E-21	Photosynthesis
	GO:0018298	8.60E-11	Protein-chromophore linkage
	GO:0009733	2.40E-09	Response to auxin
	GO:0019684	3.50E-07	Photosynthesis, light reaction
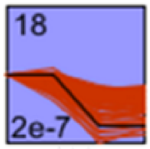 18 / 2e-7	GO:0042454	4.40E-04	Cell wall modification
	GO:0045490	4.80E-04	Pectin catabolic process
	GO:0010393	1.10E-03	Galacturonan metabolic process
	GO:0045488	1.10E-03	Pectin metabolic process
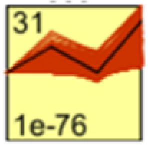 31 / 1e-76	GO:0009605	1.10E-06	Response to external stimulus
	GO:0055114	1.90E-05	Oxidation-reduction process
	GO:0031669	3.10E-05	Cellular response to nutrient levels
	GO:0015698	4.10E-05	Inorganic anion transport
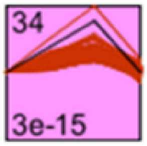 34 / 3e-15	GO:1901566	5.70E-25	Organonitrogen compound biosynthetic process
	GO:0006412	1.30E-18	Translation
	GO:0006518	3.70E-18	Peptide metabolic process
	GO:0043604	1.00E-17	Amide biosynthesis
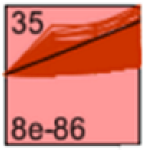 35 / 8e-86	GO:0055114	5.90E-06	Oxidation-reduction process
	GO:0035337	3.20E-04	Fatty-acyl-CoA metabolic process
	GO:1900992	3.20E-04	(-)-secologanin metabolic process
	GO:0098609	1.88E-03	Cell-cell adhesion
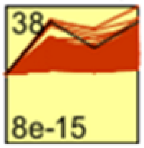 38 / 8e-15	GO:0080167	4.40E-04	Response to karrikin
	GO:0006636	5.10E-04	Unsaturated fatty acid metabolic process
	GO:0010466	1.27E-03	Negative regulation of peptidase activity
	GO:0016567	2.40E-03	Protein ubiquitination
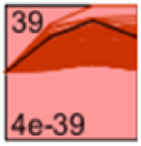 39 / 4e-39	GO:0009657	4.40E-13	Plastid organization
	GO:0042793	4.50E-08	Transcription from plastid promoter
	GO:0009451	5.30E-07	RNA modification
	GO:0044085	2.30E-06	Cellular component biogenesis

Figure S3. GO-term enrichment analysis of discrete DE/SAG expression profiles during development senescence. Analysis was performed at http://plantregmap.gao-lab.org/go.php (*P* ≤ *0.01*), and redundant GO terms were removed using REVIGO.

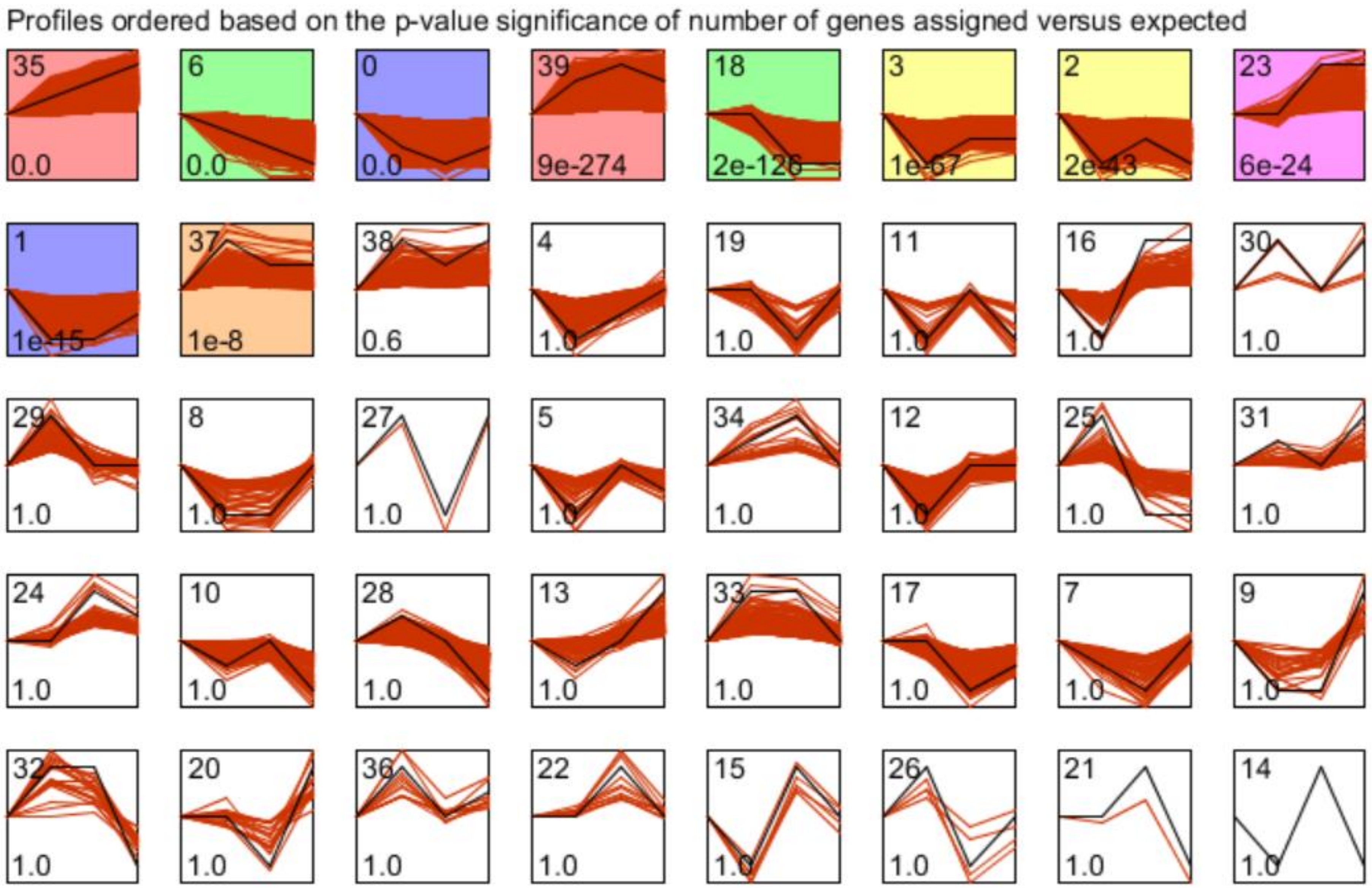

Figure S4. STEM analysis of differentially expressed genes identified during dark-induced senescence. Numbers in the top left panel represent gene profiles or clusters and numbers in the left bottom represent the P values. Profiles with P values ≤ 0.05 are considered significant. Analysis was performed using default parameters.

Profile #	GO term	P value	Description
0 (0.0)	GO:0044281	1.10E-10	Small molecule metabolic process
	GO:1901564	8.90E-10	Organonitrogen compound metabolic process
	GO:0019752	9.50E-10	Carboxylic acid metabolic process
	GO:0009628	1.70E-09	Response to abiotic stimulus
1 (1e-15)	GO:0006073	2.20E-05	Cellular glucan metabolic process
	GO:0005982	3.20E-05	Starch metabolic process
	GO:0044264	1.10E-07	Cellular polysaccharide metabolic process
	GO:0010206	1.50E-04	Photosystem II repair
2 (2e-43)	GO:0015979	1.40E-14	Photosynthesis
	GO:0019684	2.40E-09	Photosynthesis, Light reaction
	GO:0006091	8.30E-08	Generation of precursor metabolites and energy
	GO:0009767	9.80E-08	Photosynthetic electron transport chain
3 (1e-67)	GO:0044711	1.00E-06	Single-organism biosynthetic process
	GO:0042372	2.90E-06	Phylloquinone biosynthetic process
	GO:0006721	5.70E-05	Terpenoid metabolic process
	GO:0055114	6.40E-05	Oxidation-reduction process
6 (0.0)	GO:0043603	7.60E-23	Cellular amide metabolic process
	GO:0006412	1.40E-21	Translation
	GO:0009987	1.60E-14	Cellular process
	GO:0042254	2.80E-14	Ribosome biogenesis
18 (2e-126)	GO:0016310	4.00E-23	Phosphorylation
	GO:0006793	3.50E-20	Phosphorus metabolic process
	GO:0006468	1.50E-15	Protein phosphorylation
	GO:0036211	1.10E-12	Protein modification
23 (6e-24)	GO:0055065	1.40E-06	Metal ion homeostasis
	GO:2000030	3.50E-06	Regulation of response to red or far red light
	GO:0098771	9.80E-06	Inorganic ion homeostasis
	GO:0048878	3.80E-05	Chemical homeostasis
35 (0.0)	GO:1901700	6.70E-10	Response to oxygen-containing compound
	GO:0009628	8.00E-09	Response to abiotic stimulus
	GO:0009737	1.00E-08	Response to abscisic acid
	GO:0046700	6.90E-08	Heterocycle catabolic process
37 (1e-8)	GO:0006241	4.00E-04	CTP biosynthetic process
	GO:0009611	4.01E-03	Response to wounding
	GO:0034728	6.53E-03	Nucleosome assembly
	GO:0006333	7.77E-03	Chromatin assembly or disassembly
39 (9e-274)	GO:0016054	2.00E-07	Organic acid catabolic process
	GO:0046395	2.00E-07	Carboxylic acid catabolic process
	GO:0009083	1.80E-06	Branched-chain amino acid catabolic process
	GO:1901606	2.90E-06	Alpha-amino acid catabolic process

Figure S5. GO-term enrichment analysis of discrete DE/SAG expression profiles during dark treatment. Analysis was performed at "http://plantregmap.gao-lab.org/go.php", and redundant GO terms were removed using REVIGO.

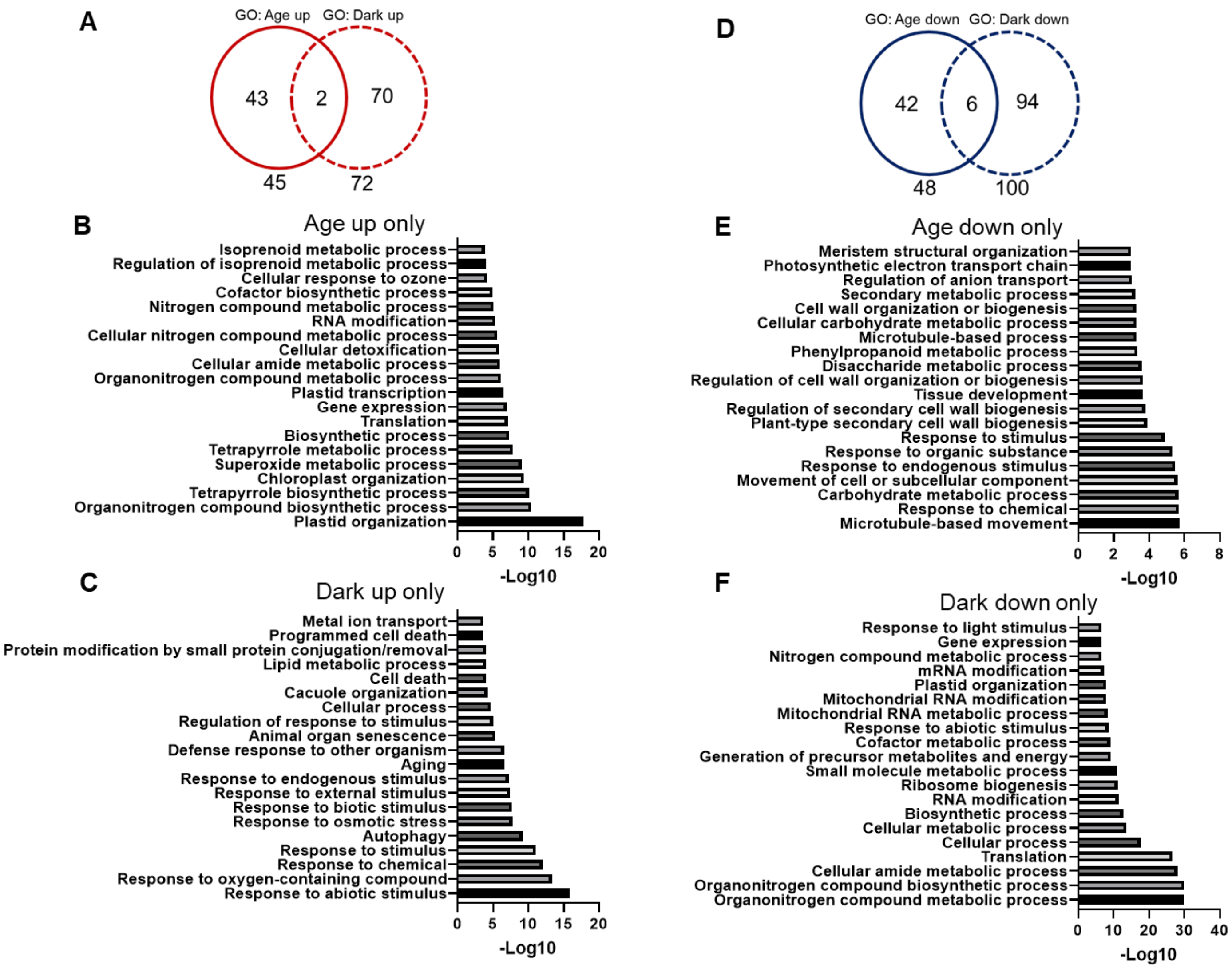

Figure S6: Comparison of GO terms specifically associated with age-dependent and dark-induced senescence. (**A)** Venn-Diagram analysis of the GO terms/biological processes encoded by DE genes that were upregulated during age-dependent (developmental) and dark-induced senescence. (**B**) Top 20 GO-terms and biological processes encoded by DE genes that were upregulated specifically during age-dependent (developmental) senescence. (**C**) Top 20 GO-terms and biological processes encoded by DE genes that were upregulated specifically during dark-induced senescence. (**D)** Venn-Diagram analysis of the GO terms/ biological processes encoded by DE genes that were downregulated during in age-dependent (developmental) and dark-induced senescence. (**E)** Top 20 GO-terms and biological processes encoded by DE genes that were downregulated specifically during age-dependent (developmental) senescence. (**F)** Top 20 GO-terms and biological processes encoded by DE genes that were downregulated specifically during dark-induced senescence. Analysis was performed at "http://plantregmap.gao-lab.org/go.php", and redundant GO terms were removed using REVIGO.

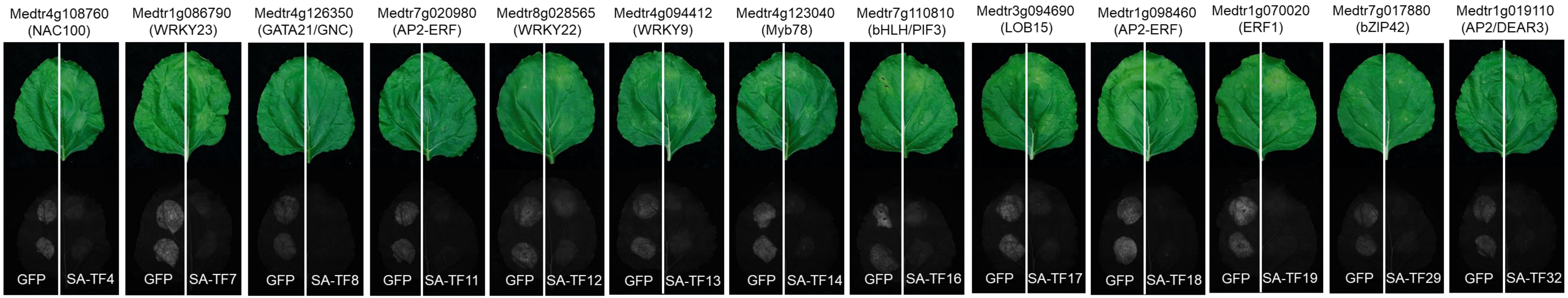

Figure S7: Transient overexpression of selected Senescence-associated transcription factors (SA-TFs) in tobacco leaves. SA-TFs that induced no senescence or mild senescence symptoms upon overexpression in tobacco leaves. Coding regions of the selected TFs were cloned in the expression vector under control of the 35S CaMV promoter and expressed in tobacco leaves using *Agrobacterium tumefacians* (GV2260). Expression of green florescent protein (GFP) was used a as positive control for transformation and negative control for senescence induction, again using the 35S CaMV promoter. Plants were phenotyped daily and photographed 6-7 days after inoculation.

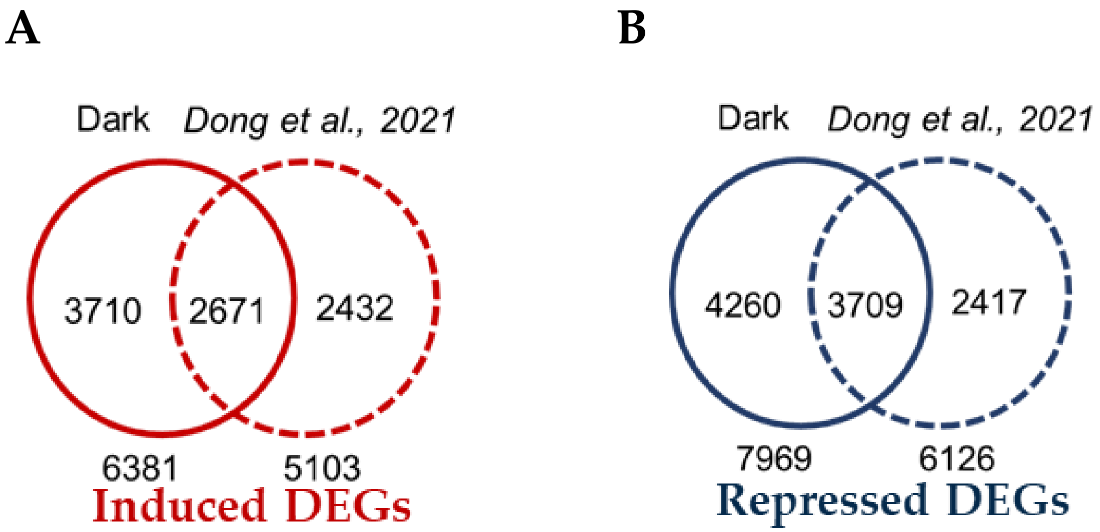

Supplementary Figure S8. Comparison of transcriptome data from dark-treated intact plants and dark-treated detached leaves in *M. truncatula*. Venn-diagram showing overlap between (A) induced and (B) repressed differentially expressed genes (DEGs) identified in our dark-induce leaf senescence transcriptome in intact plants and the dark-treated detached leaves reported by Dong *et al*., 2021.

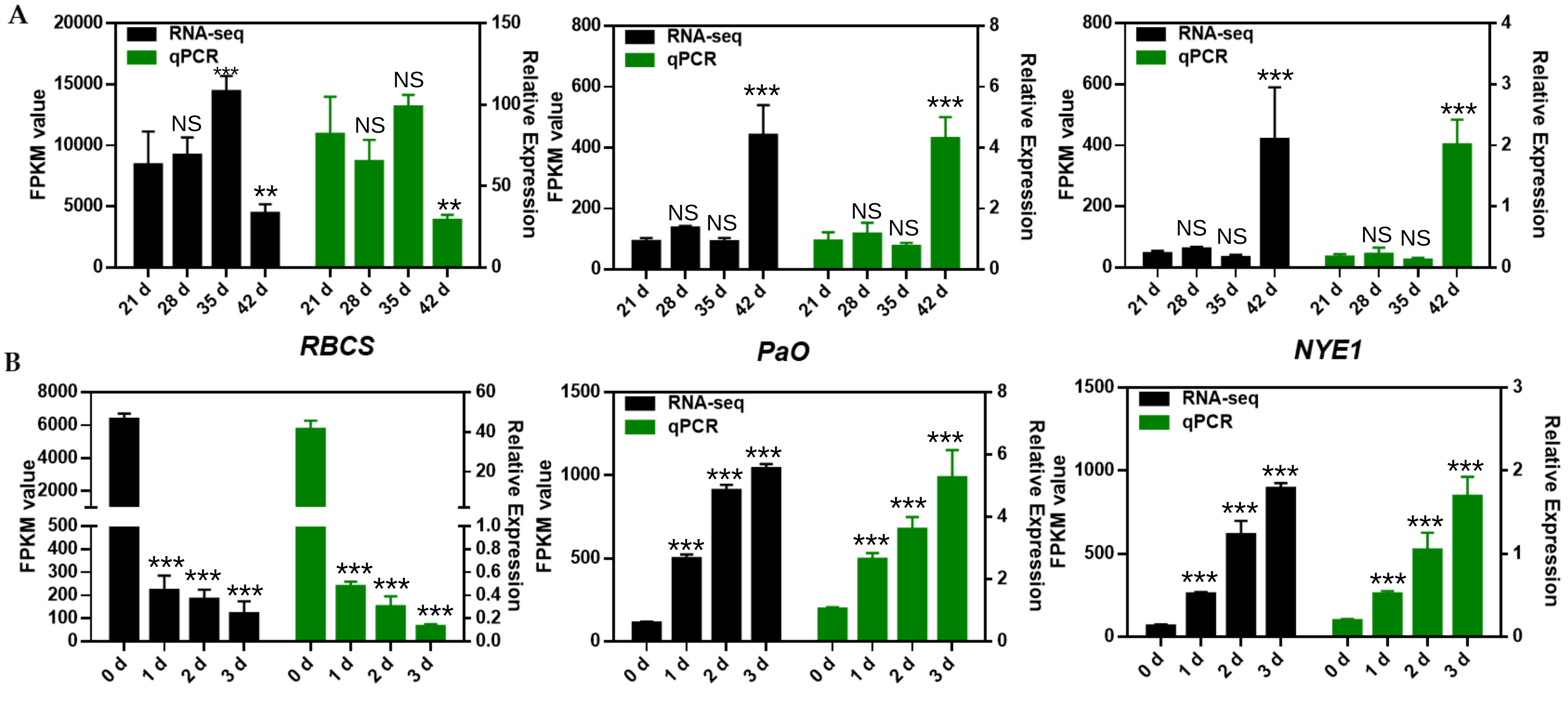

Figure S9. Confirmation of RNA-seq data by comparing the expression patterns of the *RBCS, PaO* and *NYE1* analyzed by quantitative RT-PCR with the RNA-seq FPKM values representing (A) age-dependent senescence at 21 DAS, 28 DAS, 35 DAS and 42 DAS, and (B) dark-induced senescence at 0 dD , 1 dD, 2 dD and 3 dD. The expression values of the photosynthesis-associated gene (Rubisco Small subunit, *RBCS*) and senescence-associated genes (*NYE-1* and *PaO*), shown in Figure 1C and Figure 4C, were reused here for the comparison since same RNA samples were used in qRT-PCR and RNA-seq analysis. Data in (A) and (B) represent mean values (±SD; n = 3), and were analyzed using Student's t-test (NS-Not Significant, $**P< 0.01$, $***P< 0.001$).
